# Two-dimensional pixel-level addressable mid-infrared metasurface spatial light modulator

**DOI:** 10.1038/s41467-026-75346-5

**Published:** 2026-07-07

**Authors:** Cosmin-Constantin Popescu, Maarten Robbert Anton Peters, Oleg Maksimov, Harish B. Bhandari, Rashi Sharma, Kathleen A. Richardson, Arka Majumdar, Hyun Jung Kim, Rui Chen, Khoi Phuong Dao, Luigi Ranno, Brian Mills, Dennis M. Calahan, Tian Gu, Juejun Hu

**Affiliations:** 1 Department of Materials Science & Engineering, Massachusetts Institute of Technology, Cambridge, MA USA; 2 Radiation Monitoring Devices, Inc., Watertown, MA USA; 3 The College of Optics & Photonics, Department of Materials Science and Engineering, University of Central Florida, Orlando, FL USA; 4 Department of Electrical and Computer Engineering, University of Washington, Seattle, WA USA; 5 The Department of Aerospace Engineering, KAIST, Daejeon, South Korea; 6 Draper Scholar Program, The Charles Stark Draper Laboratory, Inc., Cambridge, MA USA; 7 The Charles Stark Draper Laboratory, Inc., Cambridge, MA USA; 8 Materials Research Laboratory, Massachusetts Institute of Technology, Cambridge, MA USA

**Keywords:** Metamaterials, Metamaterials, Metamaterials, Mid-infrared photonics

## Abstract

Active metasurfaces enable dynamic control of light for applications in beam steering, pixelated holography, and adaptive optics, but demonstrations of two-dimensional electrically addressable arrays have so far been limited. Here we introduce a scalable two-dimensional architecture based on phase-change materials integrated metasurfaces and apply it to realize the first transmissive mid-infrared amplitude-only spatial light modulator. The device is fabricated through standard silicon photonic foundry processing combined with backend-of-line integration and employs multilayer backend metal interconnects to implement a crossbar addressing scheme. Each pixel is integrated with a silicon diode selector to suppress sneak-path currents, a feature essential for scaling to large arrays. The result establishes a foundry-compatible route to high-density, large-area active metasurfaces with independently tunable pixels.

## Abstract (web summary)

A mid-infrared amplitude metasurface spatial light modulator using phase change materials is demonstrated in a scalable crossbar architecture on a silicon foundry platform, allowing for high density, tunable pixel metasurfaces.

## Introduction

Active metasurfaces have emerged as a powerful platform for dynamically controlling light, enabling functions such as beam steering, holography, adaptive focusing, and on-demand wavefront shaping^1–7^. The most versatile implementations require pixel-addressable tuning, in contrast to global tuning approaches that modulate all meta-atoms simultaneously^8^. Pixel-level control is essential for two reasons. First, it allows continuous tuning of the optical response, for example, beam steering over a continuous range of angles or varifocal lenses with smoothly adjustable focal lengths, rather than being restricted to discrete switching between globally defined states. Second, it enables spatially dependent modulation of amplitude, phase, and polarization across the device aperture, which, in the limit of single-meta-atom control, can realize so-called universal optics: a reconfigurable platform capable of implementing arbitrary linear optical functions^9^. While one-dimensional electrical addressing can be realized with straightforward fan-out architectures^10–18^, extending this approach to two dimensions is only feasible for very small arrays containing a handful of independently controlled pixels (Table 1)^19–21^.

**Table 1:** Summary of representative literature reports on electrically addressed reconfigurable metasurfaces. *N/A* indicates that the corresponding information was not reported in the referenced work

Reference	Mechanism	Array architecture	Operation wavelength	Operation mode	Nonvolatile	Pixel switching time/speed	Endurance
*Nat. Commun*. **10**, 3654 (2019)^10^	Quantum-confined Stark effect in multi-quantum wells	1D fanout	~ 920 nm	Reflective	N	<1 µs	N/A
*Science* **364**, 1087-1090 (2019)^11^	Liquid crystal	1D fanout	~ 650 nm	Transmissive	N	N/A	N/A
*Nat. Nanotechnol*. **16**, 69-76 (2020)^12^	Carrier injection in indium tin oxide (ITO)	1D fanout	1.34 µm and 1.56 µm	Reflective	N	5.4 MHz (3-dB bandwidth)	N/A
*Nat. Commun*. **13**, 5811 (2022)^13^	Electromechanical actuation	1D fanout	~ 1.52 µm	Reflective	N	N/A	N/A
*Light Sci. Appl*. **11**, 141 (2022)^14^	Liquid crystal	1D fanout	460 nm – 650 nm	Reflective	N	N/A	N/A
*ACS Nano* **17**, 16952-16959 (2023)^15^	Liquid crystal	1D fanout	~ 650 nm	Reflective	N	0.8 ms (switch on) 1.2 ms (switch off)	N/A
*Nano Lett*. **24**, 9961-9966 (2024)^17^	Electrochemical modulation	1D fanout	633 nm	Reflective	N	40 ms (switch on) 55 ms (switch off)	N/A
*Nat. Commun*. **11**, 3574 (2020)^20^	Liquid crystal	2D fanout	633 nm	Reflective	N	65 ms (switch on) 40 ms (switch off)	N/A
*Nanophotonics* **11**, 2719-2726 (2022)^19^	Carrier injection in ITO	2D fanout	~ 1.3 µm	Reflective	N	N/A	N/A
*Nat. Nanotechnol*. **16**, 661-666 (2021)^43^	PCM	Single pixel	~ 1.55 µm	Reflective	Y	5 µs (amorphization) 500 ms (crystallization)	≥ 40
*Nat. Nanotechnol*. **16**, 667-672 (2021)^42^	PCM	Single pixel	~ 700 nm	Reflective	Y	520 ns (amorphization) 21 µs (crystallization)	≥ 100
*Nat. Commun*. **13**, 1696 (2022)^41^	PCM	Single pixel	~ 1.5 µm	Reflective	Y	200 ns (amorphization) 200 µs (crystallization)	≥ 50
*Nano Lett*. **25**, 7435-7441 (2025)^44^	PCM	Single pixel	~ 1.2 µm	Reflective	Y	8 µs (amorphization) 16 ms (crystallization)	6
*Adv. Mater*. **36**, 2400627 (2024)^40^	PCM	Single pixel	~ 1.5 µm	Transmissive	Y	10 µs (amorphization) 2 s (crystallization)	1250
*ACS Nano* **18**, 11245-11256 (2024)^16^	PCM	1D fanout	~ 1.52 µm	Transmissive	Y	1 µs (amorphization) 80 µs (crystallization)	≥ 1000
This work	PCM	2D crossbar with pixel-integrated selectors	~ 2.6 µm	Transmissive	Y	13 µs (amorphization) 11 ms (crystallization)	16,700

Here, we present a scalable two-dimensional (2D) electrically addressable active metasurface architecture that overcomes the scaling bottleneck, even if not reaching individual meta-atom control. The design combines backend-of-line (BEOL)-integrated phase-change materials (PCMs) with foundry-processed silicon microheater arrays addressed through multilayer backend metal interconnects, eliminating the need for complex fan-out wiring and avoiding interconnect congestion as array size increases. Importantly, each pixel incorporates a silicon diode selector to eliminate sneak-path currents, enabling independent pixel control across large arrays.

We implement this active metasurface platform to address a critical gap in spatial light modulator (SLM) technology: the absence of transmissive devices operating in the mid-infrared. Existing SLM platforms, such as liquid-crystal-on-silicon (LCoS) devices, suffer from strong absorption of liquid crystals in the mid-IR and relatively slow response times^22^. Furthermore, they can display anchoring and correlation effects that generate undulations and other defects on the scale of a few to tens of microns, competing with the desired optical output and generating potentially undesired higher order modes^23^. Digital micromirror devices (DMDs) operate only in a reflective geometry and primarily provide amplitude modulation^24^. Transmissive mid-infrared (mid-IR) SLMs remain relatively unexplored except some prior forays into numerical design modeling^25–28^, despite their advantages for compact optical layouts, cascaded optical processing, and integration with transmissive sensing or imaging systems. Our approach directly fills this gap. By leveraging the broadband transparency of PCMs^29^, the same architecture can be adapted to operate across spectral bands from the near- to the long-wavelength infrared. The PCM metasurface platform is inherently versatile, capable in principle of modulating phase, amplitude, polarization, or even optical angular momentum^30–37^. The use of nonvolatile PCMs enables persistent states without static power consumption, and reliance on foundry manufacturing provides a clear path toward continued performance scaling in pixel count, aperture size, and modulation complexity.

## Results

### 2D electrically addressable active metasurface matrix

**Fig. 1:**
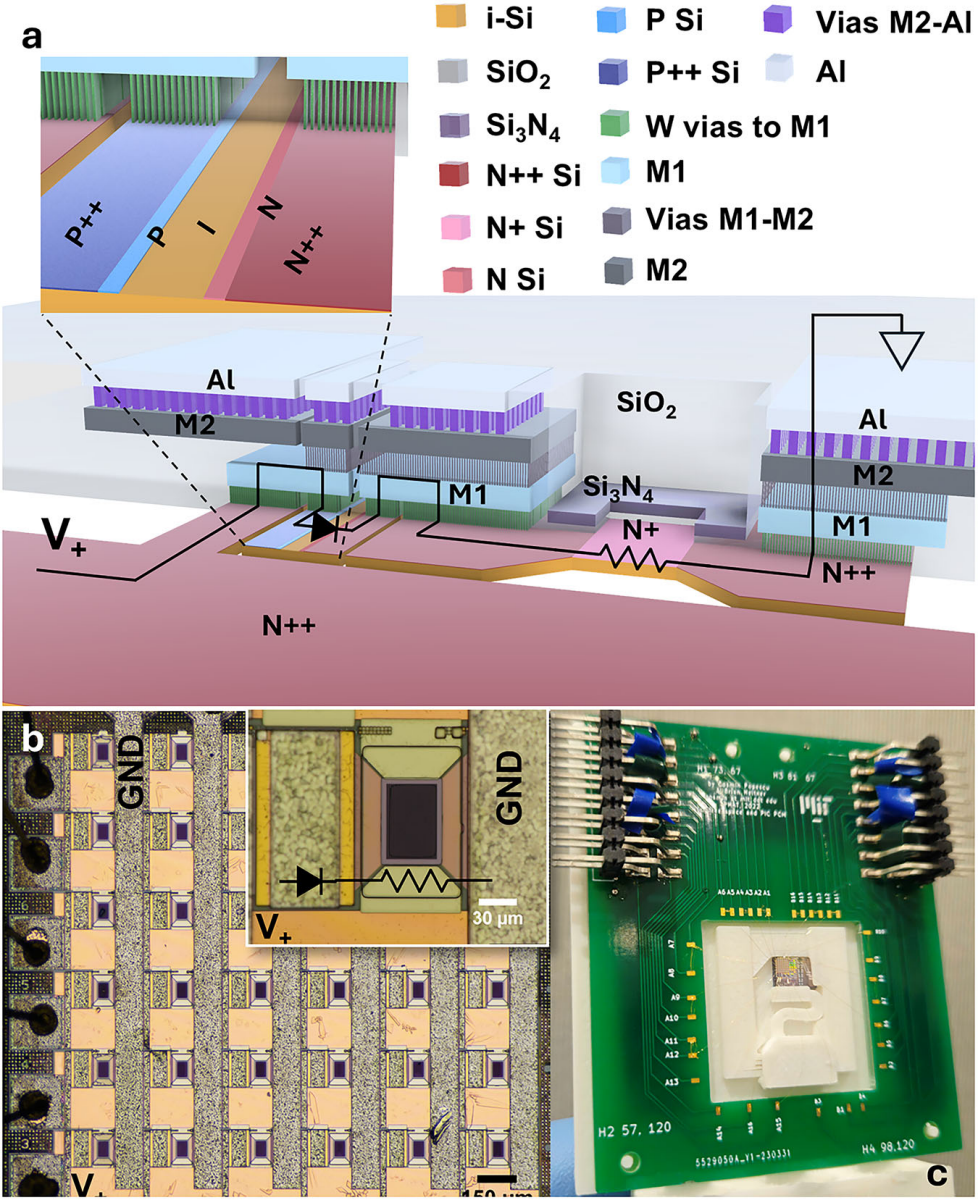
2D electrically addressable metasurface array architecture and fabricated device. **a** Schematic illustration of a single pixel showing the crossbar addressing scheme, with each crosspoint comprising a doped silicon microheater in series with a Si PIN diode selector. Two backend metal layers (M1 and M2) form the orthogonal row and column interconnects. **b** Optical micrograph of the fabricated array, with inset showing a single pixel consisting of a heater and selector. **c** Photograph of the packaged chip wire-bonded onto a custom printed circuit board carrier designed for transmissive optical measurements.

The metasurface pixel architecture is illustrated in Fig. 1a. The crossbar fabric containing the pixel array was fabricated using AIM Photonics’ standard Active Silicon process, which provides two BEOL metal layers used to form the orthogonal row and column addressing lines. At each crosspoint, the intersecting wires connect to a doped-silicon microheater in series with a Si PIN diode. The PIN diode is structured with 15 µm long P + + and N + + contact regions and 2 µm-long P and N transition regions, which are separated by an 8-µm intrinsic region, leading to a total of 42 µm length and 114 µm width. Such a PIN diode suppresses the sneaky path current, allowing for individual pixel switching with the microheaters. The microheater has 55 µm × 114 µm contact regions with 27 µm long tapers that are N + + doped, connecting to the N+ resistor region which is 40 µm × 60 µm. The signal/ high voltage V_+_ path is carried via a row on N + + silicon and on the metal level M1, while the ground is on the metal levels M2 and Al. Figure 1a highlights only the V_+_ on the doped silicon for clarity. To mitigate current-density restrictions, the number of vias between connected layers are maximized. Both the diodes and heaters are implemented using the doping levels available in the commercial AIM Photonics process. The diodes serve as unipolar selectors, blocking sneak-path currents as depicted in Fig. 1a—a critical function for large crossbar arrays, where sneak-path current otherwise increases rapidly with array size. Quantitative analysis in Supplementary Section I underscores the impact of selector integration: in an array without selectors, similar to prior demonstrations^38^, sneak-path currents already consume more power than the addressed pixel in a modest 4 × 4 array. By contrast, our selector-enabled architecture can scale to megapixel arrays with negligible parasitic power loss. The ability to seamlessly integrate pixel-level selectors is a unique feature enabled by Si foundry fabrication, alongside the inherent manufacturing scalability of the process. The nonvolatile nature of PCM further simplifies the architecture: instead of requiring a per-pixel transistor, a Si diode integrated with each microheater enables single-pixel addressing and sequential row- or column-based programming.

Following foundry fabrication, windows are opened in the BEOL dielectric to expose the microheaters. In this step, the SiN layer available as part of the AIM Photonics process serves as an etch stop, ensuring minimal damage to the underlying Si heaters. Electrical measurements performed before and after window opening confirm that heater performance is preserved (Supplementary Section II). Ge_2_Sb_2_Se_4_Te (GSST) films are deposited into these windows and then patterned into metasurface pixels via electron beam lithography and plasma etching. The pixels are encapsulated with a two-layer capping structure, which as shown in our previous study^39^ significantly improves device endurance. Optical micrographs of the fabricated array and a single pixel are shown in Fig. 1b. The completed chips are wire-bonded to a custom printed circuit board (PCB) carrier designed for transmissive optical measurements (Fig. 1c). Fabrication and packaging details are given in Methods and Supplementary Section II.

### Metasurface pixel design and characterization

**Fig. 2:**
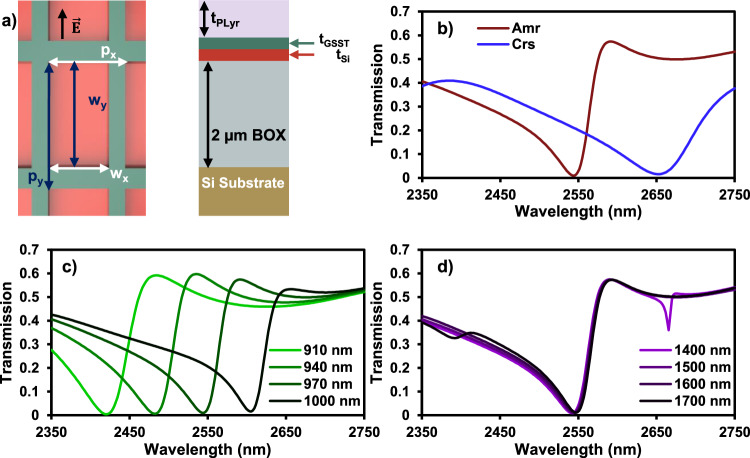
Metasurface pixel design and simulated optical response. **a** Schematic of the metasurface in-plane (left) and cross-section (right) layouts. The lattice periods are *p*_*x*_ and *p*_*y*_, with etched window dimensions *w*_*x*_ and *w*_*y*_. The thicknesses are *t*_*Si*_ = 220 nm for doped Si, *t*_*GSST*_ = 220 nm for the Ge_2_Sb_2_Se_4_Te layer, and *t*_*CL*_ = 720 nm for the capping layer. The incident light is polarized along the *y*-direction. **b** Simulated transmission spectra for *p*_*x*_ = 970 nm, *p*_*y*_ = 1600 nm, *w*_*x*_ = 768 nm, and *w*_*y*_ = 1345 nm, parameters used in the spatial light modulator devices. **c** Simulated pixel transmission spectra in the amorphous state for varying *p*_*x*_ from 910 nm to 1000 nm with *p*_*y*_ = 1500 nm, *w*_*x*_/*p*_*x*_ = 0.72 and *w*_*y*_/*p*_*y*_ = 0.8. **d** Simulated pixel transmission spectra in the amorphous state for varying *p*_*y*_ from 1400 nm to 1700 nm with *p*_*x*_ = 970 nm and the same *w*_*x*_/*p*_*x*_ and *w*_*y*_/*p*_*y*_ as in (**c**).

The metasurface pixel design, shown in Fig. 2a, is based on the principle of guided-mode resonance (GMR). A phase transition in the PCM changes the effective index of the guided mode, producing a shift in the resonance wavelength and modulating the transmission amplitude. In addition to supporting GMR, the “fishnet” metasurface design provides an array of anchoring points where the bilayer capping structure makes direct contact with the Si heater. This mechanical linkage helps mitigate PCM delamination as a leading cause of PCM device failure and thereby contributes to improved device endurance^40^. Figure 2b presents rigorous coupled-wave analysis (RCWA) simulations of the pixel transmission spectra in the amorphous (Am) and crystalline (Cr) states for light linearly polarized along the *y*-direction, showing that refractive-index contrast between the two phases modulates the GMR wavelength and generates strong optical contrast. By tuning the coupling strength to operate near the critical coupling regime, the design predicts a switching contrast ratio of 15 dB near the crystalline-state GMR wavelength of 2.65 µm. The GMR wavelength exhibits distinctive dependences on the metasurface lattice periods along the *x* and *y* axes, *p*_*x*_ and *p*_*y*_: varying *p*_*x*_ provides an effective means of tuning the resonance, whereas the wavelength is largely insensitive to changes in *p*_*y*_, as illustrated in Fig. 2c and Fig. 2d. Details of the simulation protocols and results are elaborated in Supplementary Section III.

**Fig. 3:**
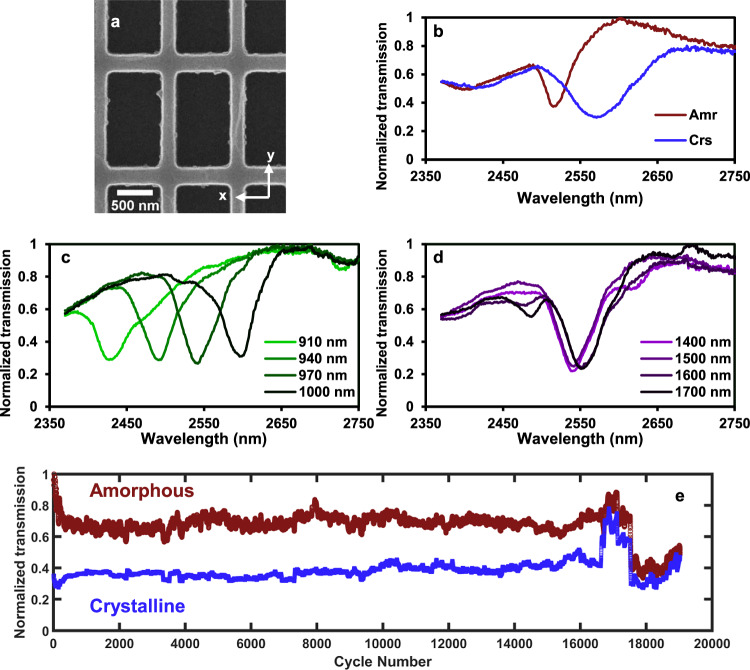
Fabricated Ge_2_Sb_2_Se_4_Te (GSST) metasurface and experimental characterization. **a** Scanning electron microscope image of the fabricated GSST metasurface after plasma etching and resist removal. **b** Measured normalized transmission spectra of a single spatial light modulator (SLM) pixel in the amorphous (Am) and crystalline (Cr) states. Normalized transmission spectra of metasurfaces with varying periods along the (**c**) *x*- and (**d**) *y*-directions, with nominal design parameters matching the simulations shown in Fig. 2c and Fig. 2d. **e** Transmission through a single SLM pixel at 2.585 µm wavelength over 19,000 switching cycles, comparing amorphous and crystalline states.

Figure 3a shows a scanning electron microscope (SEM) image of a fabricated PCM metasurface prior to capping-layer encapsulation. Figure 3b presents the measured transmission spectra of a metasurface pixel designed with the same parameters as in Fig. 2b, in both amorphous and crystalline states. Due to limitations of the experimental setup, without the possibility to obtain absolute power measurements, these results are only relative estimates based on image analysis of the images collected by the Telops camera. The experimental spectra exhibit a systematic blue shift of the GMR wavelengths relative to design values and reduced extinction ratios, which we attribute to slight deviations between the actual and assumed refractive indices of the PCM and doped-Si layers. The measured transmission contrast ratio reaches 5.1 dB at 2.585 µm wavelength and can be further enhanced by optimizing the design toward the critical coupling condition. We also characterized the transmission response of pixels fabricated with varying periods *p*_*x*_ and *p*_*y*_, with results shown in Fig. 3c and Fig. 3d. The resonance wavelength exhibits a much stronger dependence on *p*_*x*_ than on *p*_*y*_, in excellent agreement with the simulation trends in Fig. 2c and Fig. 2d. While there is some contribution from the stray light in the system (such as from the doped Si transition regions which protect the electrodes and were not covered with an absorptive aperture) accounting for this stray signal or removing it from the device would increase the contrast between the two states.

To toggle between the amorphous and crystalline states, we applied voltage pulses of 25.5 V for 13 µs for amorphization and 18 V for 11 ms for crystallization. These parameters were first optimized on a single pixel and subsequently applied uniformly across the entire array. The fact that the same set of pulse conditions produced reliable switching across all pixels without further adjustment highlights the excellent pixel-to-pixel uniformity and fabrication repeatability of the platform. Endurance testing results are shown in Fig. 3e, where the optical contrast was monitored over 19,000 switching cycles. The device maintained a stable switching response for approximately 16,700 cycles, after which delamination failure occurred. This endurance substantially surpasses previous reports for electrically switched PCM metasurface devices, including transmissive metasurface filters limited to ~1250 cycles before degradation^40^ and other metasurface implementations typically restricted to a few thousand cycles or less^41–44^. The demonstrated improvement marks a significant advance in PCM metasurface durability, with further gains anticipated through the incorporation of wetting layers to enhance adhesion and suppress delamination, and optimization in the PCM and encapsulation geometry to minimize thermally induced and phase change induced stresses.

### Metasurface 2D spatial light modulator demonstration

We implemented a 6 × 6 pixel array following the architecture in Fig. 1a, with each pixel independently addressable via electrical control. The array pitch is 400 µm, and the effective switching area per pixel is 60 × 20 µm. While the fill factor in this prototype is relatively low, it is not a fundamental limitation of the architecture. With straightforward design adjustments, the fill factor can be increased to 80–90%, values comparable to those achieved in state-of-the-art SLMs (Supplementary Section IV). Likewise, both array size and pitch can be readily optimized by leveraging the inherent scalability of the foundry fabrication process.

**Fig. 4:**
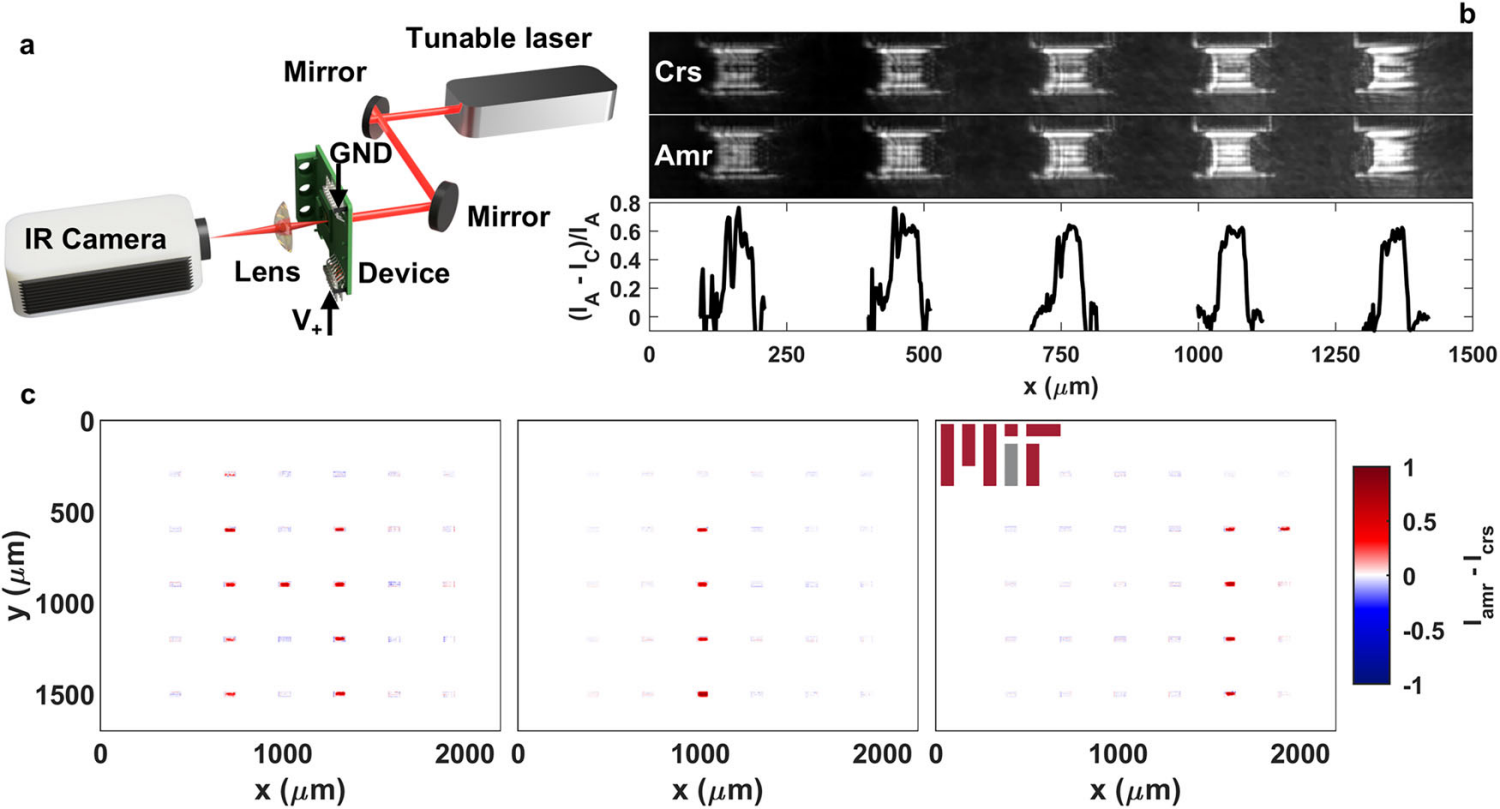
Demonstration of 2D electrically addressable transmissive mid-infrared spatial light modulator operation. **a** Schematic of the optical setup for back-illuminated transmissive imaging of the device. The electrical control circuitry is omitted for clarity. **b** Optical images at 2.585 µm of a row of pixels configured in the crystalline (off) and amorphous (on) states. Dynamic pixel switching is shown in Supplementary Video 1. **c** Normalized transmission contrast along a line scan through the center of the pixels. Here, the normalized contrast is defined as (*I*_*A*_ – *I*_*C*_) / *I*_*A*_, where *I*_*A*_ and *I*_*C*_ denote the transmission intensities in the amorphous and crystalline states, respectively. **d** 2D pixel transmission maps of the 6 × 6 array, generated by subtracting an image with the selected pixels programmed to display the letters “M,” “I,” and “T” in the crystalline (off) state from a baseline image with all the pixels in the amorphous (on) state. The corresponding dynamic array reconfiguration is presented in Supplementary Video 2.

The SLM array was characterized using the setup shown in Fig. 4a, with further details provided in Methods. Measurements were performed at a wavelength of 2.585 µm. Figure 4b compares two images of the array in which an entire row of metasurface pixels was set to the “on” and “off” states, respectively. The corresponding line-scan profile of the transmission contrast in Fig. 4c shows minimal pixel-to-pixel variation in either state, underscoring the uniformity of the device. Light transmission through doped-silicon regions without PCM films produces a bright static background surrounding the active switching areas of each pixel; this artifact can be readily eliminated in future implementations by adding a metal mask to block the background. Figure 4d presents transmission maps obtained by subtracting a baseline image with all pixels in the crystalline (“off”) state from images in which selected pixels were programmed to display the letters “M,” “I,” and “T”, emulating the MIT logo shown in the inset. Supplementary Videos demonstrate dynamic switching of these programmable patterns. These results highlight the capability for on-demand, spatially reconfigurable control of the array.

## Discussion

This work establishes and integrates multiple advances in active metasurface technology. It represents an experimental demonstration of a transmissive mid-IR SLM, a realization of a 2D electrically addressable active metasurface with a scalable architecture (as opposed to fan out architecture), and a report of a PCM-based metasurface fabricated using a standard silicon foundry process with full BEOL integration. These advancements are significant not only as technical milestones, but because the foundry platform inherently enables features critical to scalability: seamless integration of diode selectors at the single-pixel level, multilayer BEOL metals for crossbar routing without congestion, and multiple doping levels for designing large-area, efficient heaters, all with the uniformity and reproducibility required for consistent pixel performance. Although the present prototype is a small array with relatively large pitch and modest fill factor, the underlying architecture is directly extensible to high-density arrays. Additionally, the demonstration of extended endurance enabled by the choice and optimization of capping materials and other advances, are readily scalable, suggesting promise in the fabrication of larger arrays for a range of optical functionality. Specifically, the usage of tungsten plugs from a foundry run, tapered regions avoiding current crowding and separating the electrodes from the high temperature areas, and increased landing area for the capping contributed to the increased switching resilience of the heaters themselves. Larger windows in the PCM structure also likely enable better adhesion of the capping and reduced delamination risk, albeit this was insufficient to completely prevent it. We therefore anticipate that, with straightforward design refinements, the technology will rapidly evolve to deliver performance comparable to state-of-the-art near-infrared SLMs, as supported by our projections in Supplementary Section IV.

While the present demonstration establishes multiple performance benchmarks for PCM-based metasurfaces, several aspects of the prototype leave room for improvement. First, although our device already exceeds previously reported endurance values for electrically switched PCM metasurfaces, it still falls short of the lifetimes achieved by existing SLM technologies and the requirements of many practical applications. This limitation is not intrinsic to PCMs: for example, optical switching in Ge_2_Sb_2_Te_5_ has achieved endurance of 10^8^ cycles^45^. The comparatively lower endurance of electrically switched devices is generally attributed to greater non-uniformity and localized heating, but our recent work (to be published) has demonstrated over 10^7^ cycles in PCM-integrated waveguides using electrically driven heaters. We anticipate that incorporating designs such as inverse-designed microheaters with improved thermal uniformity^46^ can deliver similar or superior endurance in metasurface platforms. Greater thermal uniformity is expected to improve the switching uniformity and usable active area of the device, without requiring excessive voltage application that can degrade the metasurface due to excessive temperatures. These changes can also account for the metal apertures needed to block stray optical signal which would have higher thermal conductivity.

Second, while GSST offers broadband transparency in both phases extending to 20 µm^29^, practical operation is constrained by absorption in the buried oxide and doped-silicon heater. The former introduces significant loss beyond ~7.5 µm, which could be mitigated by backside oxide removal (at the expense of mechanical stability) or replacement with lower-loss dielectrics such as SiN. Losses from doped Si can be reduced by lowering doping concentrations (albeit at the cost of higher driving voltages) or by engineering the guided mode to minimize spatial overlap with the heater. These strategies suggest a viable route to extending the platform across the full long-wave infrared band.

Third, switching speed is ultimately limited by the heater thermal time constant, and can be engineered to reach the microsecond regime for both amorphization and crystallization, faster than LCoS and competitive with or superior to DMDs. However, the passive-matrix addressing used here restricts frame rates, as programming proceeds row-by-row or column-by-column. Realizing the full potential of fast PCM pixels will require pixel-level transistor integration for active-matrix addressing, an advance that our foundry-compatible platform is uniquely positioned to achieve. In this work, the switching speed is limited by the crystallization speed of GSST. During the endurance testing, to prevent overheating of the device, a 30 s wait time was used for the device to cool down via air convection. The chip was suspended in a plastic enclosure, with only minimal contact area between the chip and the enclosure. On average, the heat dissipated during a cycle for 18 V pulses passing 35 mA is around 7 mJ, almost all the energy being just the crystallization part, as the time for crystallization is 3 orders of magnitude larger than for amorphization. The addition of a heat sink would vastly increase the heat extraction and allow increasing the switching speed, with commercial heat sinks for similar sizes of the chip being expected to remove heat on the order of 1 W.

In sum, the platform demonstrated here lays the groundwork for a new class of scalable, pixel-addressable active metasurfaces across the mid-IR and other spectral regimes. By combining BEOL-integrated PCMs, foundry-fabricated microheaters, and integrated selectors or transistors within a crossbar architecture, we establish a manufacturing pathway that is directly compatible with high-volume silicon photonics production. This convergence of scalability, broadband operation, and nonvolatile programmability opens opportunities well beyond amplitude-only modulation. While we focused on GSST in this work due to its good balance of optical contrast and low loss in the mid-IR, the platform is PCM agnostic and other compositions can be chosen based on the required design specifications such as operational wavelength or switching time. With suitable metasurface designs, the same architecture can be adapted for phase, polarization, or orbital angular momentum control, enabling complex wavefront engineering across large apertures. In the longer term, the ability to approach single–meta-atom addressability offers a route toward reconfigurable “universal optics”—devices capable of implementing arbitrary linear optical transformations on demand. Coupled with advances in endurance, pixel density, and active-matrix driving, such metasurfaces could form the basis of compact, high-speed, and multifunctional systems for applications spanning beam steering, adaptive imaging, free-space communications, and dynamic scene projection.

## Methods

### Device fabrication and packaging

The BEOL device fabrication process is depicted in Fig. S1. Foundry-fabricated dies were first mounted on 4-inch Si carrier wafers to facilitate handling during processing. Each die consisted of a 220-nm moderately doped Si layer with heavily doped n^++^ contact wings on a 2-µm buried oxide (BOX). To define the heaters, ~5 µm of AZ nLOF 2035 resist was spin-coated, prebaked at 113 °C for 60 s, exposed in an MLA Heidelberg 150 at 375 nm with a dose of 500 mJ/cm^2^, post-exposure baked at 113 °C for 60 s, and developed for ~85 s in AZ 300 MIF. The patterned resist was ultraviolet (UV) flood-exposed and hard-baked at 125 °C for 10 min to improve resilience during subsequent etching. The chips were then mounted with Santovac oil onto 8-inch Si carrier wafers for thermal conduction and etched in two 5-min steps in an AMAT Precision 5000 system. After this etch, the resist was stripped and a second lithography step defined regions to expose the underlying nitride. The chips were immersed in buffered oxide etch (BOE) until a uniform coloration indicated that the nitride was reached. A third lithography step was then performed to expose the moderately doped Si heater region while protecting the n^++^ wings. In this step, 220 nm of SiN_x_ and 100 nm of SiO_2_ were etched away.

Following heater definition, a negative resist mask was patterned for rectangular GSST pads extending over the heater region. GSST thin films were prepared using thermal evaporation from a single Ge_2_Sb_2_Se_4_Te source. Bulk starting material was synthesized using a standard melt quench technique from high-purity (99.999%) raw elements^47^. 220 nm of GSST was deposited by thermal evaporation using a custom-designed system (PVD Products, Inc.) at a base pressure of 2 × 10^-6 ^Torr with a deposition rate of ~ 1 nm/s. Details of the deposition protocols are discussed elsewhere^48^. Lift-off was performed overnight in N-methyl-2-pyrrolidone (NMP). For metasurface patterning, 500 nm of ZEP 520 A was spin-coated and baked at 180 °C for 2 min, followed by a conductive ESpacer 300 layer. Windows were written using electron beam lithography, compensating for the ~5 µm topography between the heater surface and surrounding regions. After development in ZED 50 N for 90 s, the GSST in the exposed windows was etched in a SAMCO 230iP ICP-RIE with 25 standard cubic centimeters per minute (sccm) SF_6_ and 15 sccm Ar at 0.5 Pa, using 100 W bias and 50 W inductively coupled plasma (ICP) power. Etching was performed in short steps with intermittent inspection to ensure complete removal of the GSST without over-etching into the Si heaters. The hardened ZEP mask was stripped by overnight soaking in NMP, followed by gentle sonication.

Encapsulation of the GSST metasurface was performed in two stages. First, 20 nm of Al_2_O_3_ was deposited by atomic layer deposition (ALD) at 150 °C in a Veeco Savannah 200. A thick encapsulation layer was then added by reactive sputtering in an AJA ATC Orion 5 system, with a base pressure of 2.2 × 10^−6 ^Torr. Two Si targets were powered at 100 W each, with 7 sccm Ar and 5 sccm N_2_ at 3 mTorr, yielding ~700 nm of SiN_x_. Windows were opened back to the contact pads using photolithography and a standard SiN_x_ etch recipe in the SAMCO 230iP. To suppress background transmission through oxide regions outside the heaters, apertures were deposited by electron beam evaporation of 20 nm Ti / 120 nm Pd using an AJA ATC tool. Pd was chosen for its high melting point and low coefficient of thermal expansion, ensuring robustness under thermal cycling.

Finally, chips were wire-bonded to custom PCBs with an MEI 1204D bonder. Packaged devices were secured in custom 3D-printed holders designed to allow direct backside optical illumination during characterization.

### Optical simulation

The metasurface optical response was modeled using RCWA implemented with the open-source MATLAB® package RETICOLO V8^49^. Simulations were performed at normal incidence with the incident polarization aligned along the *y*-direction, and illumination applied from the backside through the Si substrate. The simulated stack comprised the Si substrate, a 2-µm buried BOX layer, a 220-nm Si device layer, and a 220-nm GSST fishnet metasurface. The GSST was encapsulated by 20 nm of Al_2_O_3_ and further overcoated with 700 nm of SiN_x_, with air as the top cladding.

### Device characterization

The devices were characterized using the setup shown in Fig. 4a. The chip was illuminated using a tunable Firefly M2 laser source (Firefly-IR-LP-A-BB-I) with pulse duration <10 ns and linewidth <10 cm^-1^, which averaged out the Fabry-Perot interference of the silicon substrate. For spectral measurements, the wavelength was swept in 1-nm steps with a 0.1 s dwell time. The sweep was manually synchronized with a MATLAB routine that simultaneously collected images from a Telops IR SPARK M150 camera operating in radiometric temperature mode, scaled between 18 °C and 100 °C for better image sensitivity, except for the unimpeded laser illumination for which a setting of 18 °C to 135 °C to avoid pixel saturation. The lower threshold was chosen to be 18 °C to avoid stray signal from the laboratory environment. The images were collected on an 8-bit depth and normalized to a range of 0 to 1 (with 1 corresponding to 255 from the camera). The sample image was focused onto the camera, and the relative positions of the chip and camera were adjusted to achieve the desired magnification. Images were collected during the spectral sweep, and the pixel counts/intensity in the regions of interest were averaged. A separate spectral sweep was performed with the laser hitting the camera directly, without any sample on the pathway. The averaged values for the devices were divided by the values for the unimpeded laser sweep and normalized to the highest value within the data set to show relative trends. During the endurance testing, the average time per cycle was around 30 seconds to allow sufficient cooldown for the sample in absence of any heat sink, considering that the chip was suspended in a 3D printed polylactic acid enclosure with a small support structure to not impede the optical signal.

## Supplementary information

Supplementary Information

Description of Additional Supplementary File

Supplementary Video 1

Supplementary Video 2

Transparent Peer Review file

## Data Availability

The data that support the findings of this study are available on Zenodo at 10.5281/zenodo.20563555^50^.
