# Supplementary material for: Two-dimensional pixel-level addressable mid-infrared metasurface spatial light modulator
Source: Nat Commun. 2026 Jul 7;17:8393. doi: 10.1038/s41467-026-75346-5 (PMC13473556; doi:10.1038/s41467-026-75346-5)
Supplement: Supplementary file 1 [file 41467_2026_75346_MOESM1_ESM.pdf]

Supplementary Information

Two-Dimensional Pixel-level Addressable Mid-infrared Metasurface Spatial Light Modulator

Cosmin-Constantin Popescu[1], Maarten Robbert Anton Peters[1], Oleg Maksimov[2], Harish Bhandari[2], Rashi Sharma[3], Kathleen A. Richardson[3], Arka Majumdar[4], Hyun Jung Kim[5], Rui Chen[1], Khoi Phuong Dao[1], Luigi Ranno[1], Brian Mills[1,6], Dennis Calahan[7], Tian Gu[1,8], and Juejun Hu[1,8,*]

[1]Department of Materials Science & Engineering, Massachusetts Institute of Technology, Cambridge, MA, USA
[2]Radiation Monitoring Devices, Inc., Watertown, MA, USA
[3]The College of Optics & Photonics, Department of Materials Science and Engineering, University of Central Florida, Orlando, FL, USA
[4]Department of Electrical and Computer Engineering, University of Washington, Seattle, WA, USA
[5]The Department of Aerospace Engineering, KAIST, Daejeon, South Korea
[6]Draper Scholar Program, The Charles Stark Draper Laboratory, Inc., Cambridge, MA, USA
[7]The Charles Stark Draper Laboratory, Inc., Cambridge, MA, USA
[8]Materials Research Laboratory, Massachusetts Institute of Technology, Cambridge, MA, USA

*Corresponding author email: *hujuejun@mit.edu*

This Supplementary Information comprises the following Sections:

I. Quantifying sneak-path current suppression: the selector advantage

II. BEOL integration process and characterization

III. Optical and thermal modeling of PCM metasurface

IV. Improving PCM SLM fill factor

Section I – Quantifying sneak-path current suppression: the selector advantage

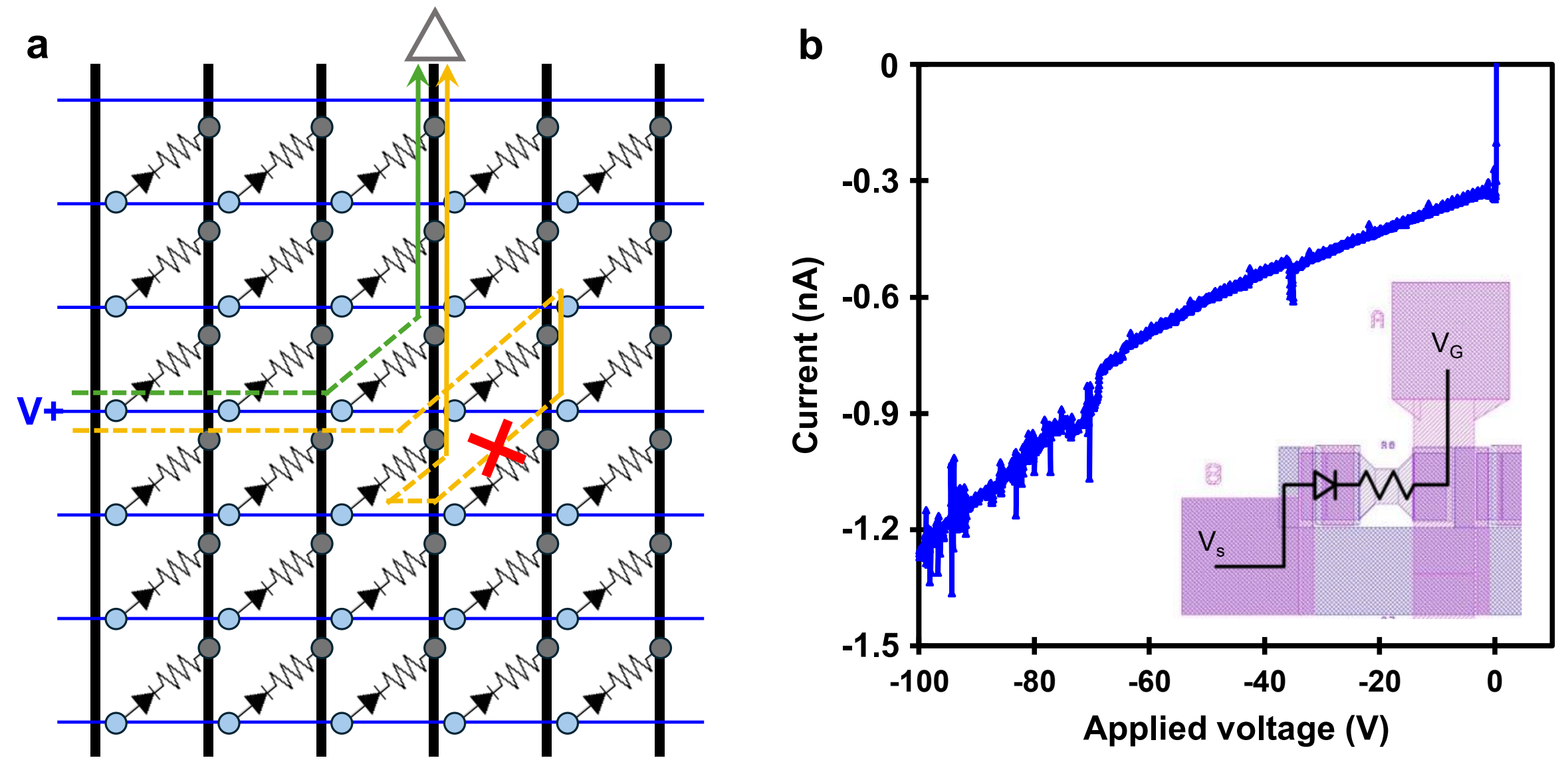

Fig. S1. Selector functionality in the crossbar architecture. (a) Schematic of the crossbar array architecture with pixel-integrated selectors. The diodes block undesired current flow along half-selected and unselected paths, thereby suppressing sneak-path currents (illustrated by yellow lines). (b) Measured reverse-bias I-V characteristic of a single SLM pixel, showing sub-nanoampere leakage over a wide voltage range. The inset shows the pixel mask layout with a superimposed circuit diagram, illustrating the PIN diode selector connected in series with the resistive microheater. Adapted from [1].

Here we consider the scaling of the SLM array size n for an $n \times n$ architecture, as schematically illustrated in Fig. S1a. The primary limitation on array size arises from sneak-path currents, which increase with n and contribute to parasitic power consumption. Without loss of generality, we assume a simplified diode model characterized by a fixed forward resistance R_f and a fixed reverse-bias saturation current I_s. From experimental measurements of the reverse-bias characteristics of a pixel (Fig. S1b), we extracted $I_s \sim 0.4$ nA under a crystallization bias of 18 V. This leakage is negligible compared with the forward-bias drive current of ~ 35 mA through the addressed pixel (Fig. S4b), corresponding to a current suppression ratio exceeding seven orders of magnitude.

Next, we apply Kirchhoff's current law to derive the total sneak-path current under the present assumptions. One selected row is biased at the crystallization voltage $V = 18$ V, one selected column is grounded at zero bias, and all remaining rows and columns are left floating. By symmetry, all floating rows share the same potential V_x, and all floating columns share the same potential V_y. Each floating row must deliver current to the selected column at zero bias through a forward-biased diode. To satisfy current balance on that floating row, it must in turn receive current via reverse leakage from the floating columns, which requires $V_y > V_x$ so that the non-addressed diodes remain reverse-biased. We therefore obtain:

$$(n-1)I_s = \frac{V_x}{R_f} \tag{1}$$

Similarly, each floating column receives current from the selected row through a forward-biased diode and then "leaks" it to the floating rows via reverse-biased diodes. Applying Kirchhoff's current law at a floating column gives:

$$(n-1)I_s = \frac{V - V_y}{R_f} \tag{2}$$

We then have:

$$V_x = (n-1)I_s R_f \tag{3}$$

and

$$V_y = V - (n-1)I_s R_f \tag{4}$$

It is straightforward to verify using experimental data that the following condition holds in the SLM array even for very large n (e.g., 10^6), yielding $V_x \sim 0$, $V_y \sim V_+$ and hence $V_y > V_x$:

$$V \gg 2(n-1)I_s R_f \tag{5}$$

The analysis indicates that for any given pixel that is programmed "on", there are a total of $(n - 1)^2$ possible sneak-current paths, each contributing I_s. The total sneak-path current is thus given by $(n - 1)^2 I_s$.

Based on experimentally measured parameters ($I_s \sim 0.4$ nA and forward-bias drive current of ~35 mA), we then plot in Fig. S2 the signal-to-leakage ratio (equivalent to the "read margin" commonly used in the memory community), defined as the ratio of the power delivered to the addressed pixel under a crystallization pulse to the total parasitic power dissipated through all $(n - 1)^2$ possible sneak-path leakage channels, as a function of array size n. The analysis shows that parasitic power loss due to sneak-path currents remains below a few percent of the pixel power consumption even for large arrays (e.g., 2,048 × 2,048), owing to the high current suppression ratio of the integrated PIN selectors. These results indicate that the proposed SLM architecture can robustly scale to large array sizes without significant efficiency penalties from sneak-path leakage.

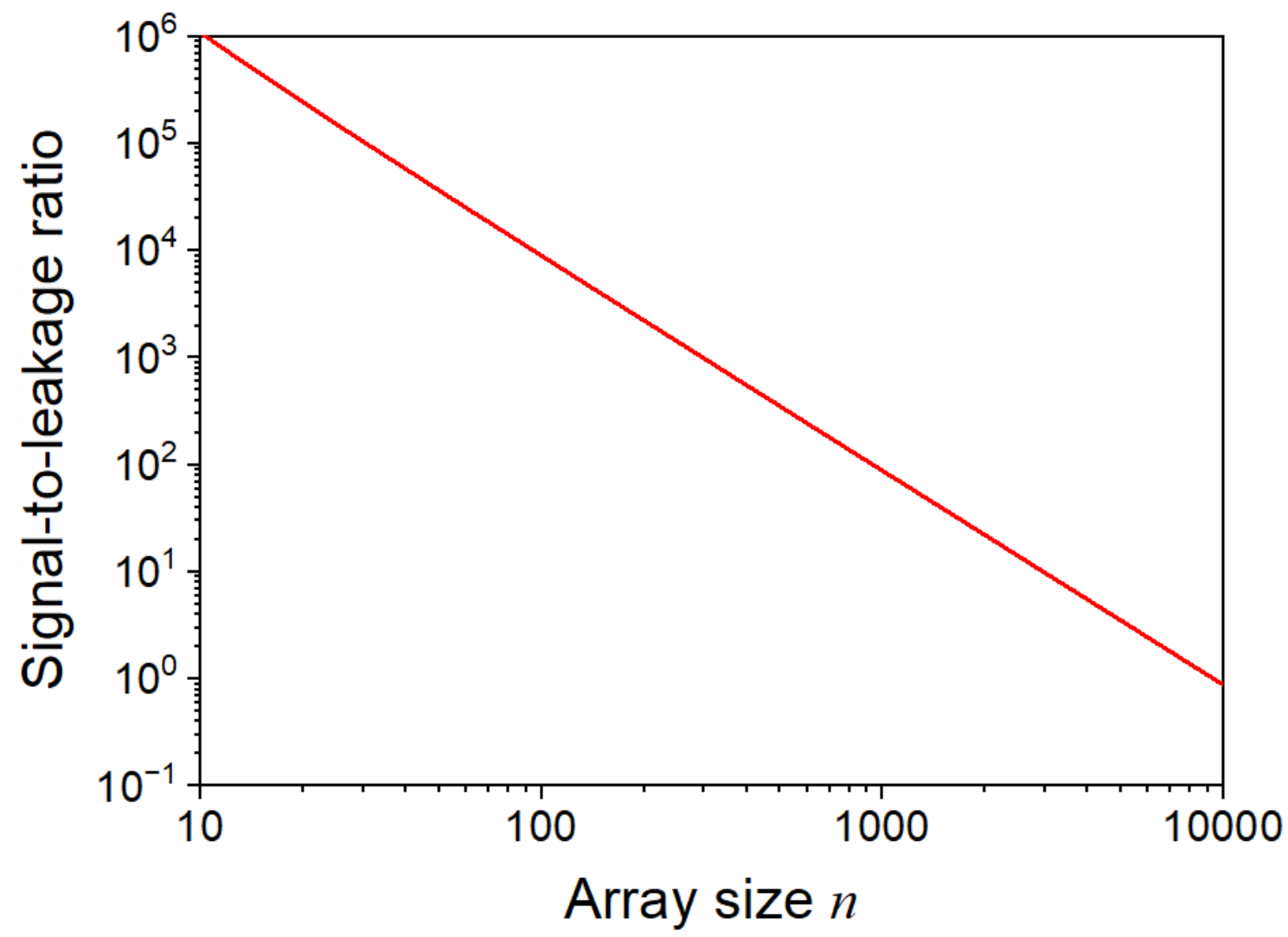

Fig. S2. Calculated signal-to-leakage ratio as a function of array size n, based on the measured forward-bias drive current and reverse-bias leakage current of a single SLM pixel. The ratio quantifies the margin between the desired pixel current and the cumulative sneak-path current summed over all possible leakage paths in an $n \times n$ array.

The same Kirchhoff's current law framework applies to crossbar arrays without selector integration, as in prior reports[2]. Assuming that each pixel behaves as a resistor R_f independent of current direction, we can still solve for the floating-node potentials V_x (rows) and V_y (columns). A floating row connects to the selected column at zero bias through a single resistor, resulting in a

current V_x / R_f leaving the row. It also connects to (n -1) floating columns at V_y, each contributing a current (V_x - V_y) / R_f. Applying Kirchhoff's current law to the floating row then gives:

$$\frac{V_x}{R_f} = (n-1)\frac{V_y - V_x}{R_f} = 0 \Rightarrow nV_x - (n-1)V_y = 0 \tag{6}$$

Similarly, a floating column connects to the selected row at V_+ through a single resistor, giving a current (V - V_y) / R_f leaving the column. It also connects to (n -1) floating rows at V_x, each contributing a current (V_x - V_y) / R_f. We therefore have:

$$\frac{V - V_y}{R_f} = (n-1)\frac{V_y - V_x}{R_f} \Rightarrow V + (n-1)V_x - nV_y = 0 \tag{7}$$

Solving the two equations yields:

$$V_x = \frac{n-1}{2n-1}V \tag{8}$$

$$V_y = \frac{n}{2n-1}V \tag{9}$$

Total power drawn from the source is:

$$P_{tot} = V\left[\frac{V}{R_f} + (n-1)\frac{V - V_y}{R_f}\right] = \frac{V^2}{R_f}\left[1 + \frac{(n-1)^2}{2n-1}\right] \tag{10}$$

The addressed pixel consumes power:

$$P = \frac{V^2}{R_f} \tag{11}$$

Therefore, the signal-to-leakage ratio is:

$$\frac{P}{P_{tot} - P} = \frac{2n-1}{(n-1)^2} \tag{12}$$

Even for a modest array size of $n = 4$, the signal-to-leakage ratio falls to 0.78, indicating that sneak-path current already dissipates more power than the addressed pixel.

Section II – BEOL integration process and characterization

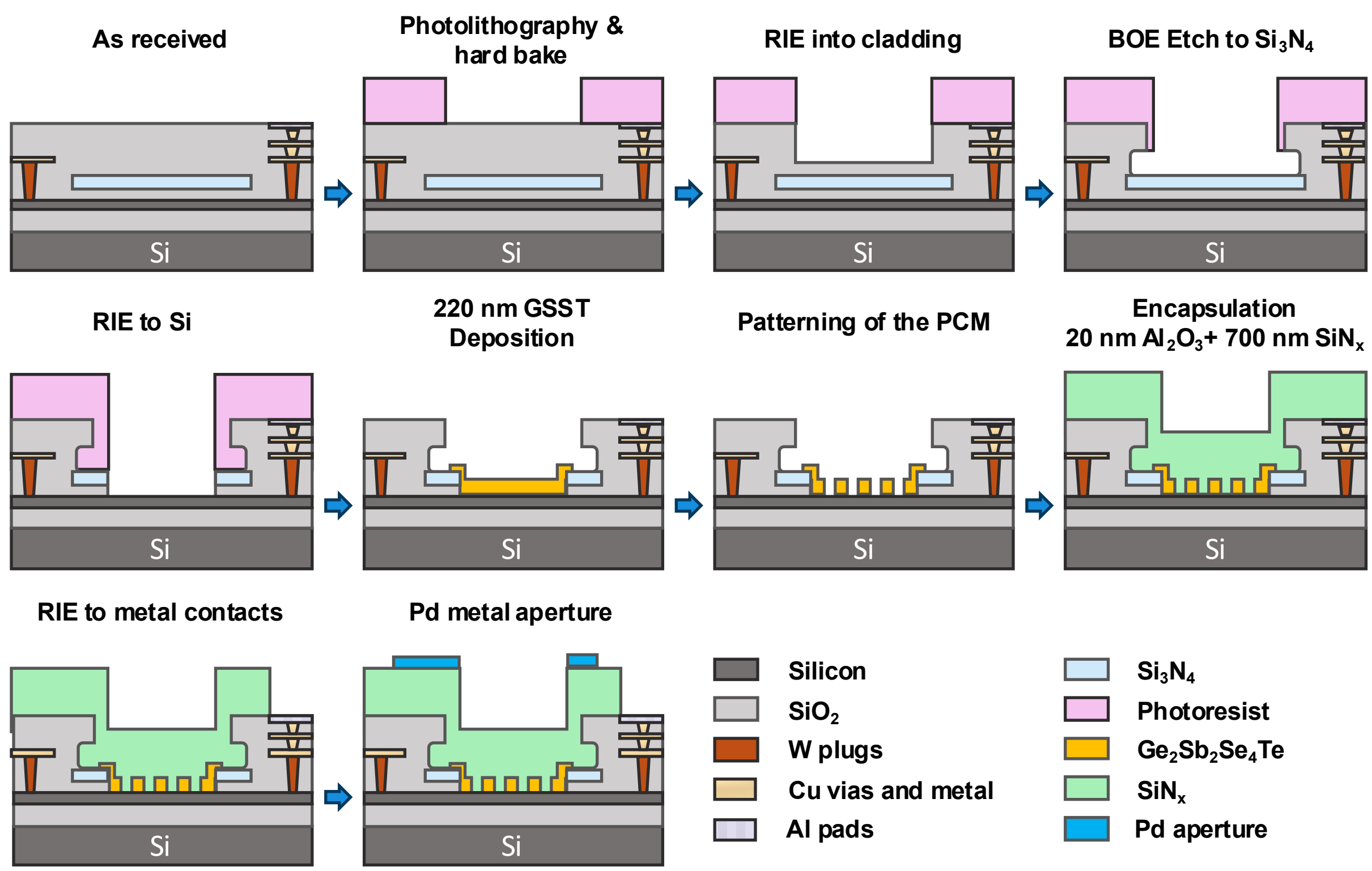

Fig. S3. Schematic illustration of the fabrication sequence used to integrate PCM metasurface arrays onto foundry-fabricated chips. Adapted from [1].

Fig. S3 schematically illustrates the fabrication process flow for BEOL integration of PCM metasurfaces onto the foundry-fabricated chips. Additional process details are provided in the Methods section. In the standard Base Active PIC process offered by AIM Photonics, a SiN layer is positioned above the silicon-on-insulator (SOI) device layer, originally intended as a passive waveguide layer. In our approach, this SiN layer was repurposed as a wet-etch stop in a three-step etching sequence, ensuring that the BOE etch reliably terminates at the SiN. This leaves a thin SiO_2 layer above SOI, which was then removed using a short, timed RIE step. Compared to a single deep etch through several microns of dielectric to directly expose the SOI heaters, this hybrid approach provides significantly improved process control and minimizes the risk of damaging the underlying SOI layer.

That our three-step etching process preserves microheater quality is verified in Fig. S4, which compares the measured current-voltage (I-V) responses of microheaters before and after BEOL integration. The negligible change confirms that the integration steps do not degrade heater performance. Moreover, the current measured at the same bias across different heaters within an array varies by less than 1%, underscoring the excellent device-to-device uniformity provided by the foundry process. This high level of consistency allowed us to employ a single set of pulse parameters for programming all pixels in the array, eliminating the need for pixel-by-pixel calibration that is often required for in-house fabricated heater devices.

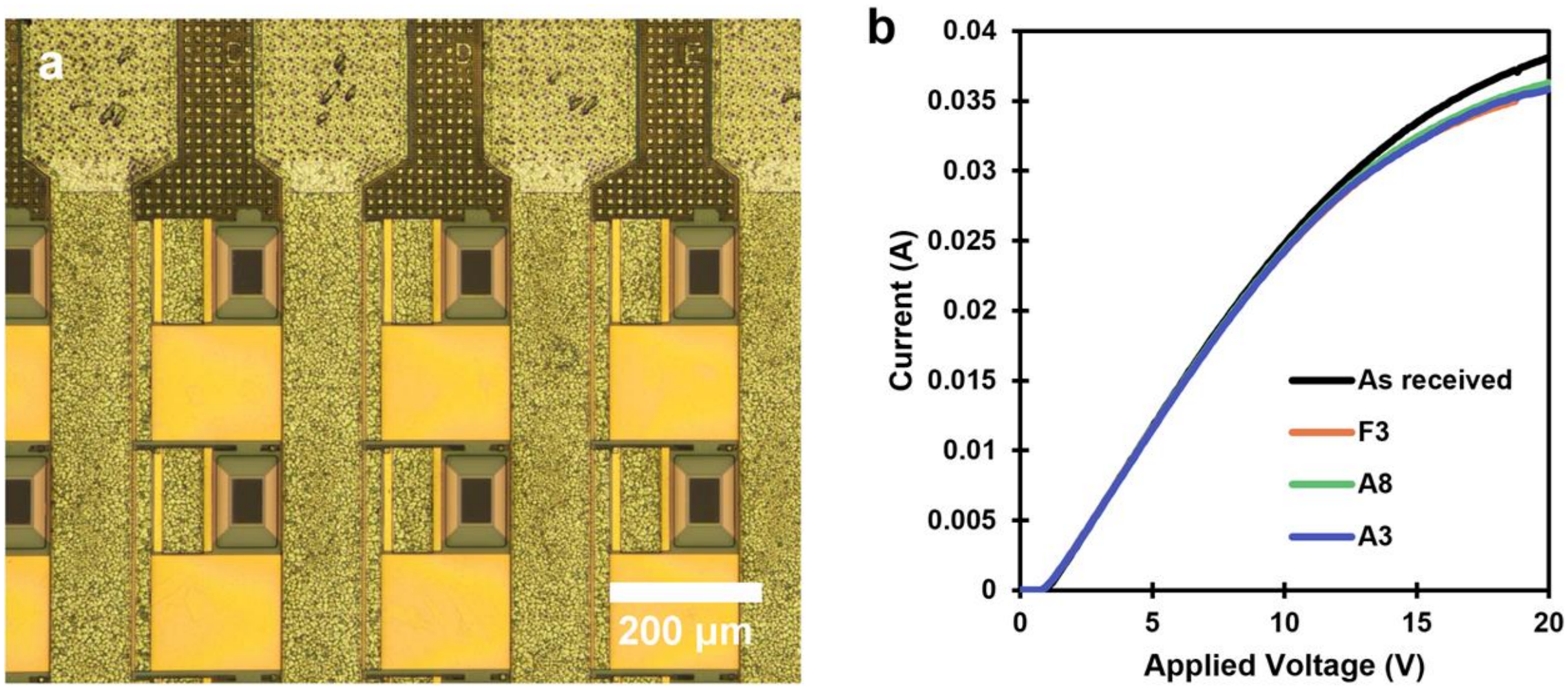

Fig. S4. Electrical characterization of Si microheaters before and after BEOL integration. (a) Top-view optical micrograph of the chip following BEOL integration of the PCM metasurfaces. (b) I-V characteristics of representative microheaters. The black curve corresponds to a measurement on an as-received foundry-fabricated chip, while the colored curves show results from three different heaters (F3, A8, and A3) after the full BEOL integration process. The close agreement confirms that the BEOL integration steps preserve heater performance and further highlights the excellent device-to-device consistency enabled by the foundry process.

Section III – Optical and thermal modeling of PCM metasurface

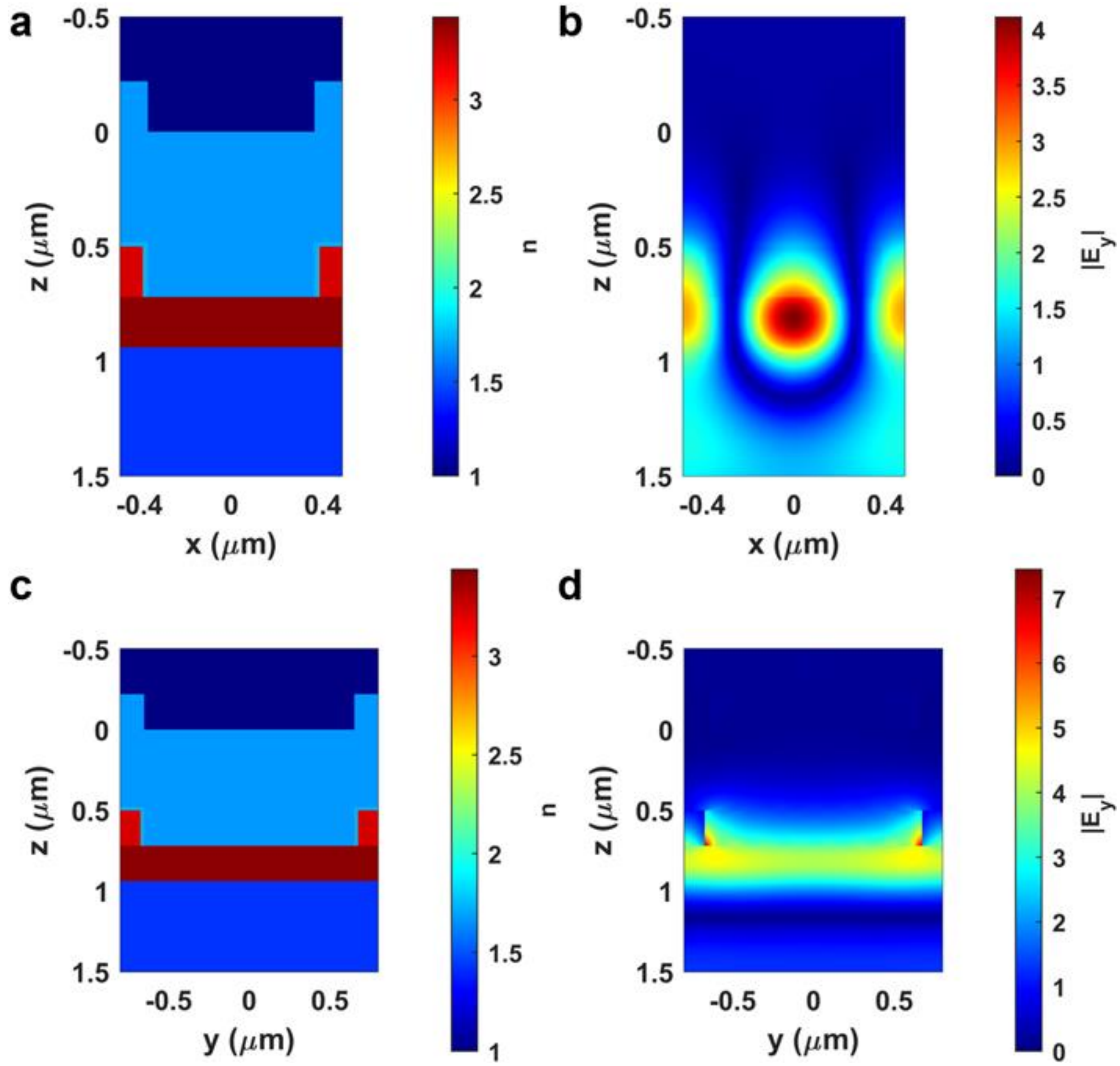

Fig. S5. Optical modeling of the PCM metasurface. (a, c) Cross-sectional refractive index profiles of the metasurface unit cell in the (a) *x*-*z* and (c) *y*-*z* planes. (b, d) Simulated electric-field amplitude distributions in the (b) *x*-*z* and (d) *y*-*z* planes through the center of the unit cell. The structure is modeled in the amorphous state and illuminated by normally incident light polarized along the *y*-direction at a wavelength of 2.585 μm.

Fig. S5 presents optical simulations of the PCM metasurface. The field maps reveal strong confinement within the SOI layer and surrounding high-index contrast interfaces, with pronounced field enhancement localized near the etched apertures. This behavior is characteristic of guided-mode resonance (GMR), where the periodic structure couples incident plane waves into laterally guided modes that interfere with the transmitted wave. The resulting resonant interaction produces the sharp transmission features observed in both simulation and experiment, underscoring the role of the fishnet metasurface (functioning here as a 2D meta-grating supporting GMR) in enabling efficient optical modulation.

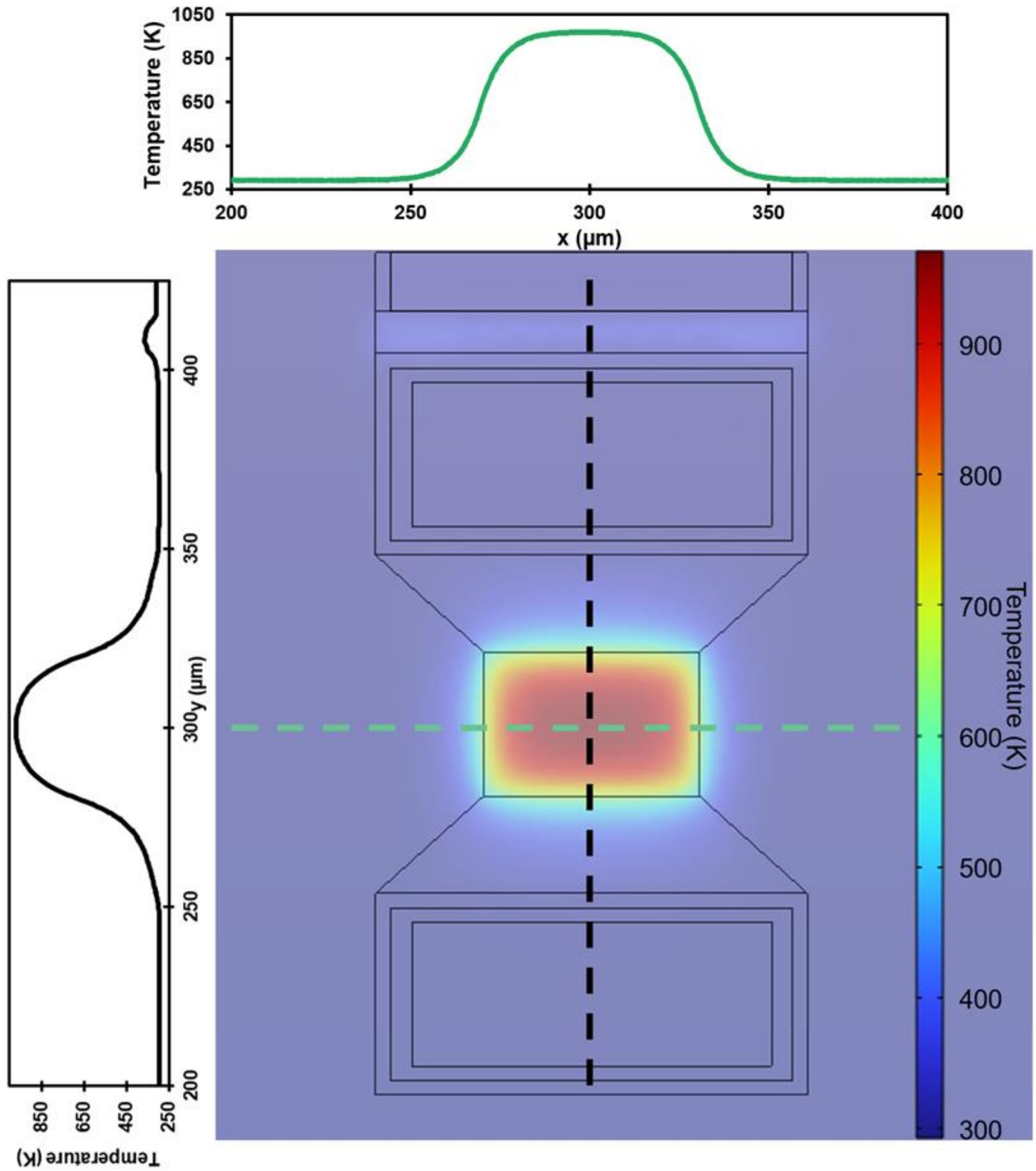

Fig. S6. Simulated peak temperature distribution across a single pixel under an amorphization pulse. The central panel shows the in-plane 2D temperature map, with horizontal and vertical line cuts marked by dashed lines. The top plot presents the temperature profile along the horizontal cut, while the left plot shows the profile along the vertical cut.

Fig. S6 and Fig. S7 show the simulated peak temperature distributions across a single metasurface pixel under amorphization and crystallization pulses, respectively. The simulations were carried out using the COMSOL Multiphysics package following protocols described previously[3]. In both cases, the thermal profile indicates that the dominant heat generation occurs within the doped-Si microheater, producing a broad and uniform high-temperature region sufficient to drive phase transitions in the PCM. A smaller contribution arises at the forward-biased PIN diode selector due to carrier injection and series resistance, leading to local temperature rises of ~ 68 K under the amorphization pulse and ~ 23 K under the crystallization pulse. This heating

is negligible compared to the heater-induced thermal load and does not affect the overall switching characteristics. Furthermore, because the selector is laterally displaced from neighboring pixels, its localized heating does not contribute appreciably to thermal crosstalk. These results confirm that the thermal design is governed by the engineered heater response, while the integrated selector provides electrical isolation without compromising pixel-level switching performance.

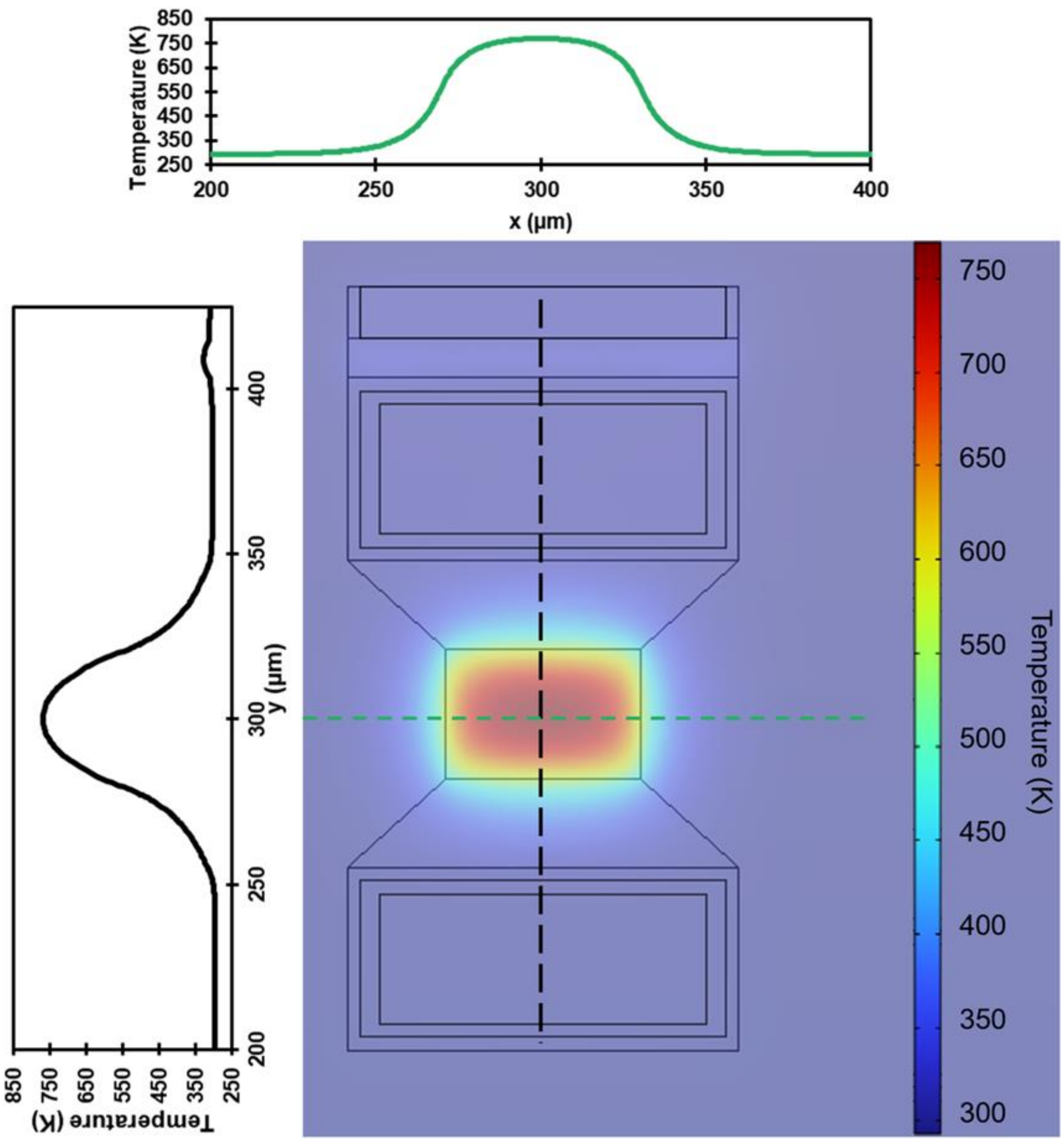

Fig. S7. Simulated peak temperature distribution across a single pixel under a crystallization pulse. The central panel shows the in-plane 2D temperature map, with horizontal and vertical line cuts marked by dashed lines. The top plot presents the temperature profile along the horizontal cut, while the left plot shows the profile along the vertical cut.

Section IV – Improving PCM SLM fill factor

In this section, we focus on quantitative projections for the fill factor of SLMs based on the PCM metasurface platform introduced in this work. While the main text has established the experimental feasibility of 2D electrically addressable mid-IR operation, practical deployment in imaging, beam steering, and communication systems requires arrays with significantly higher fill factors than those demonstrated in the current prototype. Here, we examine how fill factor can be improved through straightforward design variations and assess the practical limits that constrain further scaling.

In reflective SLM configurations, the interconnect metal lines can be routed beneath the SOI microheaters (for example, using a substrate-inverted fabrication process[4]) so the fill factor is not constrained by wiring but instead primarily by thermal crosstalk. As shown in Fig. S7, the simulated temperature decreases rapidly to below 200 °C, which is below the crystallization temperature of GSST (250 °C)[5], at a lateral distance of ~ 3.6 μm from the heater edge. This thermal profile therefore defines the minimum safe spacing between neighboring pixels in a reflective array.

In transmissive configurations, by contrast, interconnect metal lines must be routed through the gaps between neighboring pixels. The fill factor is thus limited by both the maximum tolerable current density in the metal lines (before electromigration or breakdown occurs) and thermal crosstalk between adjacent heaters. Although the amorphization pulse is shorter and reaches higher peak temperatures, the crystallization process represents the primary constraint on interconnect reliability. For Cu interconnects typically used in BEOL stacks, current densities up to 8 MA/cm^2 at 300 °C have been sustained for several hours[6], corresponding to > 10^6 PCM switching cycles. With a typical top-layer metal thickness of 3 μm, this corresponds to a maximum allowable current of approximately 240 mA per micron of line width.

To estimate the resulting fill factor limit, we next analyze the current scaling behavior of the PCM pixels. Following the model by Aryana *et al.*[7], the microheater power consumption scales linearly with heater area down to sizes of ~ 10 μm, below which deviations arise as heating of surrounding regions becomes increasingly significant. In this analysis, the driving voltage for crystallization (18 V) and heater geometry are fixed, keeping the electrical resistance size-independent. Consequently, the heater current scales inversely with its area and can be extrapolated from the I-V characteristics shown in Fig. S4b. This relationship allows prediction of the minimum interconnect width required to deliver the crystallization current, which, together with the thermal crosstalk condition, determines the maximum attainable fill factor in transmissive PCM SLMs.

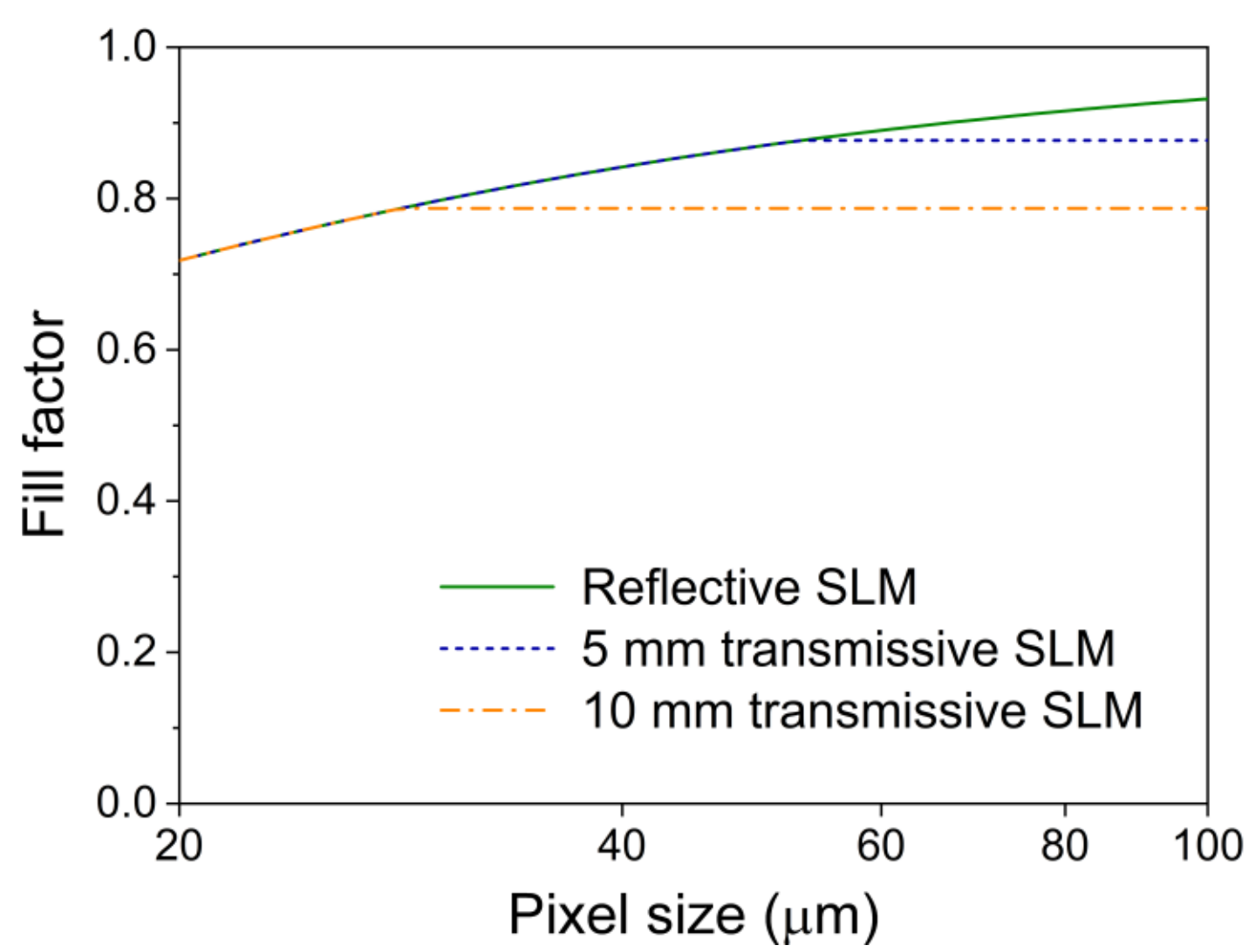

Fig. S8. Calculated fill factor as a function of pixel size for reflective SLMs and transmissive SLMs with 5-mm and 10-mm aperture sizes.

Using the parameters discussed above, we computed the fill-factor limits for reflective and transmissive SLMs, as shown in Fig. S8. For the transmissive configuration, results are plotted for two representative aperture sizes of 5 mm and 10 mm. An extreme-case scenario was assumed in which the interconnect metal lines must carry the total current required to simultaneously crystallize all pixels in a single row (or column). In reflective SLMs, the fill factor is governed solely by thermal crosstalk, which imposes a fixed minimum pixel spacing of 3.6 μm independent of pixel size. The scale-invariant behavior of the temperature decay length has been verified in our prior work[8].

For both transmissive SLM configurations, the fill factor curves exhibit two distinct regimes. At small pixel sizes, the minimum pixel spacing is determined by the thermal crosstalk constraint. As the pixel size increases, the required driving current also increases, and the maximum allowable current density in the metal interconnects becomes the dominant limitation. For larger SLM apertures (10 mm vs. 5 mm), the higher number of pixels per row or column demands proportionally wider metal lines to accommodate the total current. Overall, the analysis indicates that PCM-based SLMs can achieve fill factors in the range of 80-90% for practical aperture sizes, comparable to state-of-the-art SLM technologies operating in the near-infrared.

The above discussion is based on design parameters derived from our current prototype. Further improvements in fill factor can be pursued along several directions: refining pixel geometry to reduce the required drive current and enhance thermal confinement; lowering the drive current through reduced doping concentration in combination with higher pulse voltages; and, perhaps most effectively, adopting PCM compositions with faster crystallization kinetics, which reduce the thermal load and improve energy efficiency. In this work, we employed GSST for its low optical loss in the mid-IR. By contrast, Ge-Sb-Te (GST) alloys crystallize much more rapidly (< 100 ns)[9], requiring lower crystallization energy but introducing moderate optical loss. Importantly, the SLM architecture is not limited to chalcogenide PCMs; it can also be applied to phase-transition oxides such as VO_2, which undergoes a thermally triggered metal-insulator transition with hysteresis.

One additional note on methods to increase the fill factor is the integration of transistors instead of the current diodes for microheater selection. From prior steady state measurements, at 26 V the system conducts 0.0414 A, and at 18 V, 0.037 A. This would mean a current density through the 220 nm thick, 60 μm wide medium doped region (the region dedicated to the metasurface) is 3.14 $\times 10^9$ A/m^2 (or 0.314 MA/cm^2) and 2.80 $\times 10^9$ A/m^2 (or 0.280 MA/cm^2), respectively. The peak current values are higher on the microsecond scale but last only a small fraction of the pulse, contributing a relatively small amount of energy. If we take a transistor with a drive current capacity of 1 mA/μm[10], then we would need 42 μm wide transistors in place of the current diode to satisfy the steady state current drop. To account for the peak current, a potential 50% buffer may be needed, placing the transistor size at ~60 μm, comparable to the size of the current active region, making the key limit still the BEOL interconnection and their current density thresholds. The width of the required transistor gate will scale approximately linearly with the width of the microheater region. All of this analysis is presuming CMOS only solutions, but other types of selectors may also be integrated in the future, such as ovonic ones which tolerate higher current densities.

Supplementary References

1. Popescu, C.C. *et al.*, Heterogeneous integration of nonvolatile phase-change photonics. in *AI and Optical Data Sciences VI SPIE* 13375, 74-85 (2025)
2. Guo, T. *et al.* Durable and programmable ultrafast nanophotonic matrix of spectral pixels. *Nat Nanotechnol* 19, 1635–1643 (2024).
3. Zhang, Y. *et al.* Electrically reconfigurable non-volatile metasurface using low-loss optical phase-change material. *Nat Nanotechnol* 16, 661–666 (2021).
4. Ranno, L., Sia, J. X. B., Dao, K. P. & Hu, J. Multi-material heterogeneous integration on a 3-D photonic-CMOS platform. *Opt Mater Express* 13, 2711–2725 (2023).
5. Zhang, Y. *et al.* Broadband transparent optical phase change materials for high-performance nonvolatile photonics. *Nat Commun* 10, 4279 (2019).
6. Roy, A. & Tan, C. M. Very high current density package level electromigration test for copper interconnects. *J Appl Phys* 103, 093707 (2008).
7. Aryana, K. *et al.* Toward Accurate Thermal Modeling of Phase Change Material-Based Photonic Devices. *Small* 19, 2304145 (2023).
8. Dao, K. P. & Hu, J. Thermal inverse design for resistive micro-heaters. *J Appl Phys* 136, 225101 (2024).
9. Lankhorst, M. H. R., Ketelaars, B. W. S. M. M. & Wolters, R. A. M. Low-cost and nanoscale non-volatile memory concept for future silicon chips. *Nat Mater* 4, 347–352 (2005).
10. Packan, P. *et al.* High performance 32nm logic technology featuring 2nd generation high-k + metal gate transistors. in *Technical Digest - International Electron Devices Meeting, IEDM* (2009). doi:10.1109/IEDM.2009.5424253.
