# Supplementary material for: Two-dimensional pixel-level addressable mid-infrared metasurface spatial light modulator
Source: Nat Commun. 2026 Jul 7;17:8393. doi: 10.1038/s41467-026-75346-5 (PMC13473556; doi:10.1038/s41467-026-75346-5)
Supplement: Supplementary file 2 [file 41467_2026_75346_MOESM2_ESM.pdf]

The Description of Additional Supplementary Files

Supplementary video 1: Stop-motion video with a demonstration of sequential crystallization and re-amorphization of the PCM metasurfaces, one pixel at a time in a line.

Supplementary video 2: Stop-motion video with a demonstration of sequential crystallization and re-amorphization of the PCM metasurfaces writing the letters “M”, “I”, and “T”, showing individual control of the pixels in the cross-bar array and arbitrary shapes that they can produce.
