# Supplementary material for: Two-dimensional pixel-level addressable mid-infrared metasurface spatial light modulator
Source: Nat Commun. 2026 Jul 7;17:8393. doi: 10.1038/s41467-026-75346-5 (PMC13473556; doi:10.1038/s41467-026-75346-5)
Supplement: Supplementary file 3 [file 41467_2026_75346_MOESM5_ESM.pdf]

nature portfolio

Peer Review File

Two-Dimensional Pixel-level Addressable Mid-infrared Metasurface Spatial Light Modulator

Corresponding Author: Dr Cosmin-Constantin Popescu

This file contains all reviewer reports in order by version, followed by all author rebuttals in order by version.

Version 0:

Reviewer comments:

Reviewer #1

(Remarks to the Author)
In their work, Popescu and co-authors demonstrate PCM-based light modulator with single-pixel addressing enabled by backend-of-line technology from AIM Photonics. Successful implementation of electro-thermal switching of the optical elements with standard silicon foundry platform is a valuable addition to the field, and an important step towards translation of oPCMs from laboratory research to real-world technology. I recommend this paper for publication, provided the important points listed below are addressed in full.

1. The description of BEOL architecture is very fragmented and should be improved to make the manuscript more cohesive. Figure 1a is quite hard to navigate, as it has multiple similar-colored elements that are quite challenging to distinguish without prior knowledge of the device layout. I suggest the authors simplify the figure design (or make insets with a zoom-in on important elements such as diode selector) and make it more clear how the crossbar contact grid is implemented. Furthermore, references to works describing the foundry device in more detail, if there are any materials available publicly, would help.
2. The authors focus particularly on the transmissive capabilities of the device. In that context, the data in the manuscript needs to be more consistent, and be presented in a quantitative rather than qualitative manner. For example, Fig. 3 alternates between “normalized transmission” and “transmission”. What is the data normalized to? Considering the high refractive index of the silicon substrate, absolute transmission values can be quite low, but are necessary to estimate the application potential of the device. Furthermore, Fig.4 has an unlabelled colorbar. What do negative values in it imply (and why one of the pixels in the right panel seems “broken”)? Are they coming from the interference fringes observed in the laser transmission image? This interference seems to be quite strong, which makes one wonder how the spectra from Fig.3 were achieved and whether the interference has some contribution to the observed signal magnitudes. Furthermore, I would expect the spectra to be influenced by the FP interference in the silicon carrier substrate, particularly for parallel beam excitation configuration. Were they observed in the raw data and how they were processed?
3.Why does the device architecture used in the manuscript show an order of magnitude improvement in the endurance over the author’s previous work (ref 33)? As the authors claim this is a significant advance, the design solutions that led to this improvement need to be elaborated. Furthermore, the way the data is presented currently makes one wonder if the pixel from 1e is just a lucky one that happened to be more robust. The authors need to provide more statistics from multiple pixels to support their claim.
4. The paper has a very detailed outlook, which compensates to some extent for the fact that the proof-of-concept device is lacking in many of the aspects (fill-factor, transparency band, etc.). I suggest complementing this outlook with discussion on thermal-diffusion-limited operation speed of the device, and its comparison to state-of-the art SLMs.
5. Do the authors envision their “recent work” (line 208) to be published in any format before this manuscript? If not, I suggest that the reference to unpublished data is removed.

Reviewer #2

(Remarks to the Author)
The manuscript describes an electrically tuneable phase change metasurface pixel array. The authors demonstrate the feasibility of electrically switching arrays of phase change pixels which, if scaled further, could be used to develop a useful metasurface.

I think the technical achievements in this paper are worthy of publication in a Nature Comms. However, I believe the title of this work could be misinterpreted. When I read the title, I understood that each meta-atom was addressable. This assumption was then reinforced by the intro, which describe the importance of controlling individual meta-atoms to control the phase, polarisation, and amplitude of light. However, the authors have actually created an array of addressable fishnet-metasurfaces—not individually addressable meta-atoms within a metasurface. Addressable, metaatoms would be much more impactful because, in principle, the far field intensity and phase profile could be sculpted to produce a range of useful features in the far-field—such as holograms. Whereas the actual demonstration simply creates an array of absorber-transmitters. Therefore, please can the authors increase the clarity of the title, and also early on in the manuscript explain that their aim for the present work is not to control individual meta-atoms.
Prior to publication, please can the authors also address the following points:
1. At EPCOS in Sept 2025, Prof Juejun Huof also described excellent results for electrically addressable Sb2S3 (rather than GSST) pixels. Please can these results also be included in the present manuscript.

2. Line 57, the authors discuss the advantages of PCMs over liquid crystals. I think they should also discuss scalability issues of LCs (e.g. anchoring effects and LC correlation lengths being similar to meta-atoms).

3. Why did the authors choose to use doped-Si heaters? Why not use ITO or some other IR-transparent conductor?

4. Why did the authors focus on GSST—please justify its use over other competing phase change materials.

5. Why does the device fail due to delamination? How can this effect be prevented?

6. How does the cyclability depend on the set-reset voltage, current, and frequency?

7. The cycleability in Fig 3(e) looks good but it would look even better if you added some statistics. Was this just the best cycling result? Or did all the devices perform to this standard?

8. Fig 4d shows the M I T characters displayed on the image matrix. There was also a nice video uploaded showing these characters appearing. Although these images and videos clearly show the tunability of the pixel transmission, it seems there is still considerable room for improvement. I.e. the pixel uniformity for the 'off' and 'on' states seems to be an issue, and there are some rogue pixels. Moreover, the actual transparent area of each pixel seems to vary considerably. Please can the origin of these issues be described in the main manuscript, and please suggest ways to resolve them in future iterations of the device.

9. To toggle between the amorphous and crystalline states voltage pulses of 25.5 V for 13 μs for amorphization and 18 V for 11 ms for crystallization were used. What current was used for these switching events? I believe the current density will be quite high.

10. On line 228 the authors suggest that the individual pixels could be addressed with transistor selectors. And that these selectors could be integrated on the BEOL by the foundry. We agree that this would be necessary for much higher density metasurface SLMs. However, we believe that this might be easier said than done. For the typical reset current densities (see point 9), how big would the AIM-foundry-defined transistor need to be to enable the GSST pixel to reset?

11. The metasurface pixels are quite large. How would you expect the switching performance and cyclability performance of the device to scale as you reduce the pixel size down to a single meta-atom.

Reviewer #3

(Remarks to the Author)
I co-reviewed this manuscript with one of the reviewers who provided the listed reports. This is part of the Nature Communications initiative to facilitate training in peer review and to provide appropriate recognition for Early Career Researchers who co-review manuscripts.

Version 1:

Reviewer comments:

Reviewer #1

(Remarks to the Author)
In the revised manuscript, the authors satisfactorily addressed most of my comments. I recommend the paper for publication, but still suggest to add the discussion on the last point that was not properly covered in the revision.

This is connected to my questions 3 and 4; what I meant by thermal diffusion-limited operation speed is exactly what authors mentioned in their response to the question on the device cyclability (30 s dwell time was needed to address the issues of the device overheating). I suggest the authors mention what is the total dissipated power in the device operation regime and at which operation speed the issue can be addressed with a heat sink adequate for the chosen device format.

Reviewer #4

(Remarks to the Author)
The manuscript reports the realization, numerical, and experimental investigation of a pixel-level electrically addressable transmissive metasurface operating in the mid-infrared. The device is based on phase-change materials, integrates microheater-driven switching, and relies on a crossbar addressing architecture implemented in a silicon photonics platform. The device demonstrates programmable transmission patterns using a 6×6 pixel array fabricated in a foundry-compatible BEOL process, aiming toward scalable electrically programmable meta-optical devices. The work combines metasurface design, PCM electro-thermal switching, and integrated addressing circuitry to realize a proof-of-concept mid-IR programmable optical modulator.
The technical content is sound, the writing is clear, and the topic is timely and aligned with the journal's audience.

In my view, the authors have satisfactorily addressed all but one of the concerns raised during the previous review round, and the manuscript has improved accordingly. However, one concern raised in the previous review round appears only partially addressed, namely, regarding the quantitative interpretation of the optical measurements. While the authors clarified that the transmission values are derived from camera intensity measurements, the data remain non-absolute (see also question 2a below).

I have however identified a few elements that would benefit from further elaboration prior to endorsing the publication of this article.

1) The manuscript advances several claims of novelty that appear somewhat overstated when compared with the cited literature.

a. First, the authors claim the "first transmissive mid-IR spatial light modulator (SLM)" based on a metasurface. While the mid-IR transmissive implementation is indeed distinctive, prior works have already demonstrated transmissive electrically addressable PCM metasurfaces and SLM-like devices in the near-IR (e.g., Fang et al., 2024; Popescu et al., 2024). Thus, the novelty here seems primarily the spectral range (mid-IR) rather than the transmissive PCM metasurface SLM concept itself.

b. Second, the manuscript claims the "first 2D electrically addressable active metasurface with a scalable architecture." However, earlier studies have reported 2D electrically programmable metasurface arrays with pixel-level control, even if implemented with different wiring schemes (e.g., Li et al., Nat. Commun. 2020 and related works). The true distinction of the present work appears to lie in the crossbar addressing scheme with integrated selectors enabling scalability, rather than 2D electrical addressability per se.

c. Finally, the paper suggests that it is the first PCM-based metasurface fabricated using a standard silicon photonics foundry with full BEOL integration. However, foundry-compatible BEOL PCM integration has already been demonstrated for photonic devices (e.g., 10.1021/acsnano.4c00340).
d. There is at least one work investigating Pixel-level addressable SLMs for the mid-IR, 10.1364/AO.54.010698, albeit not leading to a practical realization.

To avoid priority conflicts, the authors should narrow their claims to emphasize the combination of selector-enabled crossbar addressing, transmissive mid-IR metasurface operation, and realization within a foundry-compatible BEOL platform, rather than broadly claiming first demonstrations of electrical addressability or BEOL PCM integration. Moreover, it would be useful to expand the literature review to include numerical investigations, rather than limiting it to practical implementations.

2) Several technical aspects of the manuscript would benefit from clarification or additional analysis. These points, taken individually, do not strongly affect the authors' key claims; however, addressing them would improve the quality of the work and strengthen the manuscript.

a. The reported transmission maps and contrast metrics appear to be derived from camera intensity values rather than calibrated optical transmission. Since the data are normalized to detector response rather than referenced to incident optical power, the reported contrast and uniformity cannot be interpreted as absolute transmission or efficiency. The authors may therefore consider either pursuing a calibrated transmission measurement or reference normalization; or, alternatively, explicitly framing the current results as relative estimates limited by the experimental setup.

b. The manuscript acknowledges parasitic transmission through doped-Si regions outside the active metasurface. This background signal likely contributes to the measured "on" state and may affect the reported contrast. The impact on the true optical extinction ratio and device efficiency should be quantified, or at least discussed.

c. Key performance metrics such as absolute transmission, insertion loss, and optical throughput are not reported, making it difficult to assess the practical performance of the device as a spatial light modulator (e.g. an extinction ratio of 5.1 dB has a different practical impact, whether the transmission is absolute or normalized).

d. In the demonstrated passive-matrix crossbar scheme, pixel programming appears intrinsically sequential, implying a rewrite time that scales as n2 for an n×n array. Given the reported switching times (~13 µs amorphization and ~11 ms crystallization), full-frame rewriting would become impractically slow for large arrays. Conversely (as a gedankenexperiment), fully parallel programming would require extremely large instantaneous power and heat dissipation,

potentially causing thermal cross-talk and compromising amorphization due to reduced quenching rates. A discussion of the scaling trade-offs concerning write/erase time and energy would strengthen the manuscript.

e. Since PCM switching is thermally driven, experimental characterization or simulation of thermal cross-talk between neighboring pixels would be important for evaluating large-array operation.

f. Although framed as an SLM, the demonstrated functionality is essentially pixelated amplitude modulation, without demonstration of phase control or wavefront-shaping capabilities. Although this critique may appear purely semantic, clarifying this distinction would improve the positioning of the work, as SLM is often used for amplitude, phase, and combined phase-amplitude modulation.

Version 2:

Reviewer comments:

Reviewer #4

(Remarks to the Author)
The revised manuscript presents a demonstration of a pixel-level, electrically addressable, transmissive metasurface operating in the mid-infrared. The combination of PCM-based non-volatile switching, selector-enabled crossbar addressing, and foundry-compatible BEOL integration is a notable contribution toward scalable programmable metasurfaces.

In my perspective, the authors have addressed all the concerns raised in the previous review round, and the manuscript has improved in clarity and completeness. The authors' commentary on the measurement limitation, parasitic effects, scalability, plausible device operation, and work positioning, answers to all my prior queries.

I would, however, encourage one more the authors to slightly refine the wording of the novelty claims in the initial paragraph of the Discussion/Conclusion, where multiple “first-of-their-kind” statements are presented. The current phrasing give the impression of orthogonal advances (lines 199-203), whereas the novelty lies in the combination of mid-IR transmissive operation, selector-enabled crossbar scalability, and foundry-compatible BEOL integration (as the authors remark elsewhere). A minor adjustment to reflect this integrated contribution would improve precision and avoid ambiguity. This is however a matter of clarity, rather than substance; it does not affect the significance of the results.

Overall, the manuscript is suitable for publication.

Open Access This Peer Review File is licensed under a Creative Commons Attribution 4.0 International License, which permits use, sharing, adaptation, distribution and reproduction in any medium or format, as long as you give appropriate credit to the original author(s) and the source, provide a link to the Creative Commons license, and indicate if changes were made.
In cases where reviewers are anonymous, credit should be given to 'Anonymous Referee' and the source.
The images or other third party material in this Peer Review File are included in the article’s Creative Commons license,

unless indicated otherwise in a credit line to the material. If material is not included in the article's Creative Commons license and your intended use is not permitted by statutory regulation or exceeds the permitted use, you will need to obtain permission directly from the copyright holder.
To view a copy of this license, visit https://creativecommons.org/licenses/by/4.0/

REVIEWER COMMENTS

Reviewer #1 (Remarks to the Author):

In their work, Popescu and co-authors demonstrate PCM-based light modulator with single-pixel addressing enabled by backend-of-line technology from AIM Photonics. Successful implementation of electro-thermal switching of the optical elements with standard silicon foundry platform is a valuable addition to the field, and an important step towards translation of oPCMs from laboratory research to real-world technology. I recommend this paper for publication, provided the important points listed below are addressed in full.

We thank the reviewer for the supportive comments.

1. The description of BEOL architecture is very fragmented and should be improved to make the manuscript more cohesive. Figure 1a is quite hard to navigate, as it has multiple similar-colored elements that are quite challenging to distinguish without prior knowledge of the device layout. I suggest the authors simplify the figure design (or make insets with a zoom-in on important elements such as diode selector) and make it more clear how the crossbar contact grid is implemented. Furthermore, references to works describing the foundry device in more detail, if there are any materials available publicly, would help.

We thank the reviewer for bringing up these issues. We have improved our Figure 1 to make the PIN diode region clearer, added labels for the signal and ground in the optical micrographs in Fig. 1b, and added the following text:

At each crosspoint, the intersecting wires connect to a doped-silicon microheater in series with a Si PIN diode. The PIN diode is structured with 15 μm long P++ and N++ contact regions and 2 μm-long P and N transition regions, which are separated by an 8-μm intrinsic region, leading to a total width of 114 μm. Such a PIN diode suppresses the sneaky path current, allowing for individual pixel switching with the microheaters. The microheater has 55 μm × 114 μm contact regions with 27 μm long tapers that are N++ doped, connecting to the N+ resistor region which is 40 μm × 60 μm. The signal/ high voltage V_+ path is carried via a row on N++ silicon and on the metal level M1, while the ground is on the metal levels M2 and Al. **Error! Reference source not found.**a highlights only the V_+ on the doped silicon for clarity. To mitigate current-density restrictions, the number of vias between connected layers are maximized. Both the diodes and heaters are implemented using the doping levels available in the commercial AIM Photonics process.

2. The authors focus particularly on the transmissive capabilities of the device. In that context, the data in the manuscript needs to be more consistent, and be presented in a quantitative rather than qualitative manner. For example, Fig. 3 alternates between “normalized transmission” and “transmission”. What is the data normalized to? Considering the high refractive index of the silicon substrate, absolute transmission values can be quite low, but are necessary to estimate the application potential of the device.

We are grateful to the reviewer for pointing out the inconsistencies.

All the figures in Fig. 3 should be “normalized transmission” and we have corrected that. The transmission was estimated based on image analysis from an IR camera of the switching region. We specifically put “normalized transmission” due to the difficulty to get absolute power measurements using the IR camera. As such, we normalized the camera readouts to a reference readout, where the laser illuminated the camera directly without any sample. Then the results were normalized by the highest value in the resulted value. The readouts are average pixel values from regions of interest in the captured images.

The data for Figs. 3c and 3d were normalized to the highest pixel readings among the devices, where the devices were imaged at the same magnification and illumination and the regions of interest were chosen close to the center of the devices to avoid stray signal from the electrodes or edges of the microheater regions.

To be more specific:

Without any sample on the path, the laser was swept across wavelengths and images were collected and the pixel count/intensity was calculated from averaged regions on interest (ROI). An example image is shown below. To avoid saturating the image, the bounds for the scaling of the direct laser illumination were 18 ^{o}C to 135 ^{o}C radiometric temperature on the camera.

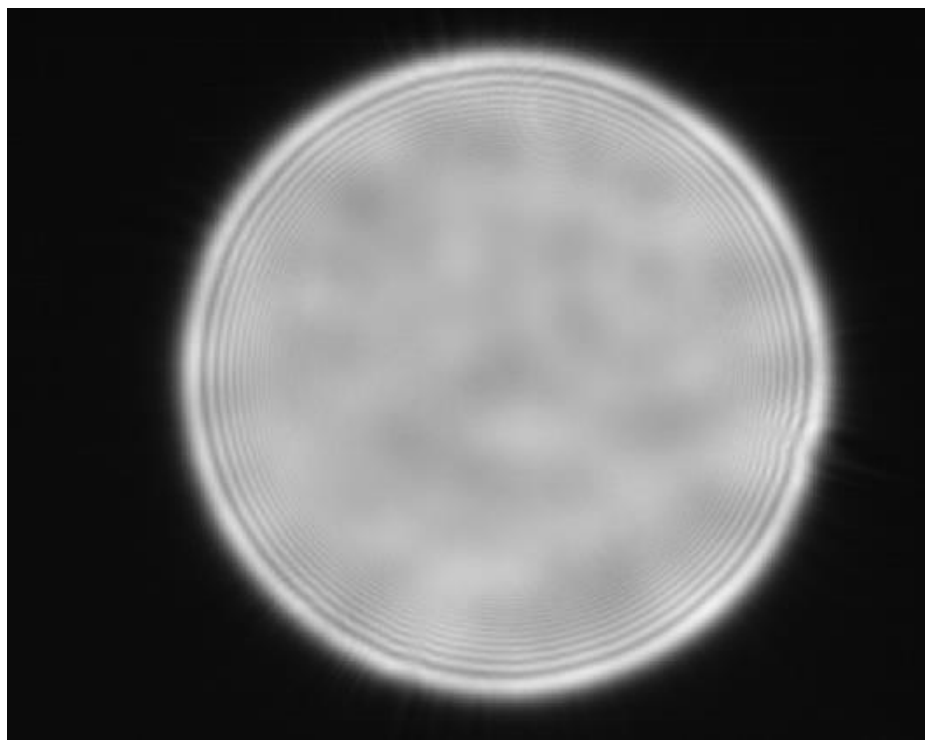

Similarly, the metasurfaces with microheaters are brought into focus and the laser is swept. For better contrast, the bounds are set to 18 °C to 100 °C for the radiometric temperature scale. Example images close to the resonance of the respective metasurfaces are shown below:

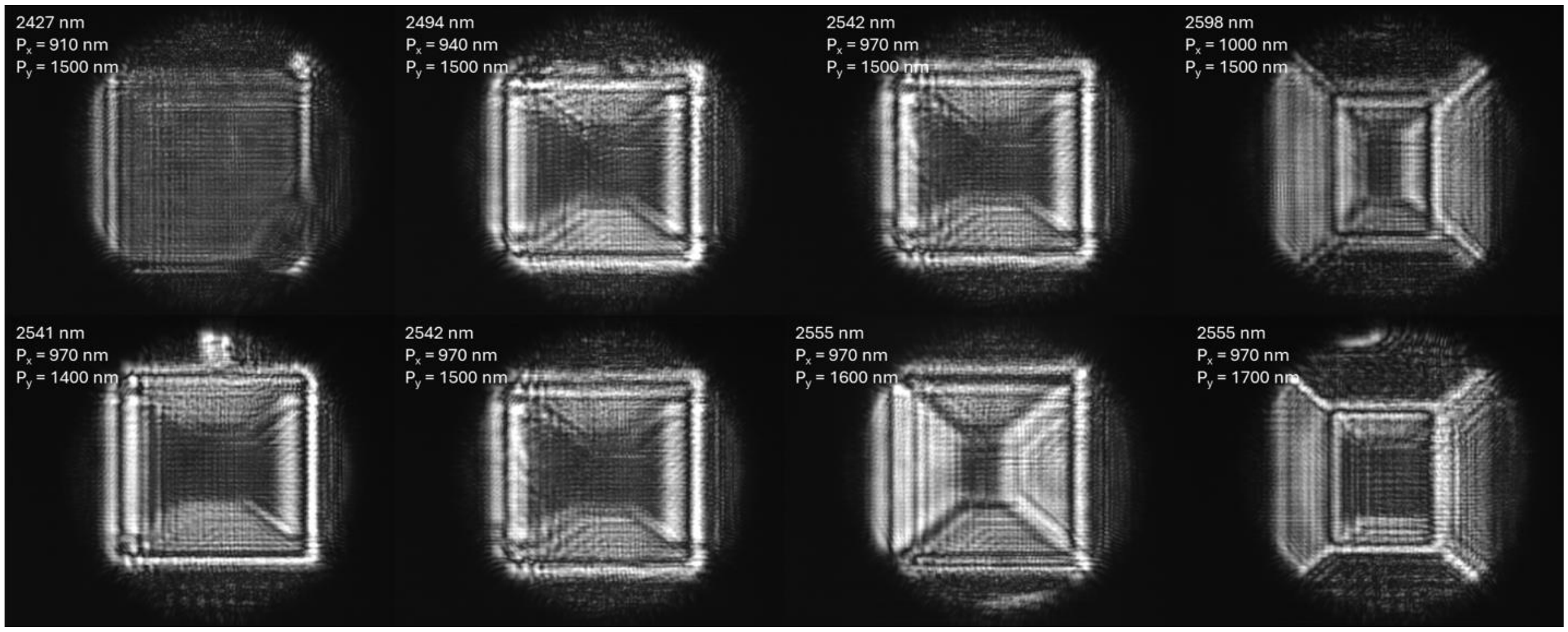

The ROI is chosen in these images to avoid the scattered light from other features such as the regions without silicon (above and below the heater) and the side regions where the metasurface ends and the signal increases.

The average values from the pixel readings (raw signal) are plotted below. Note for instance the local increase in signal due to higher power from the laser at around 2675 nm and 2725 nm.

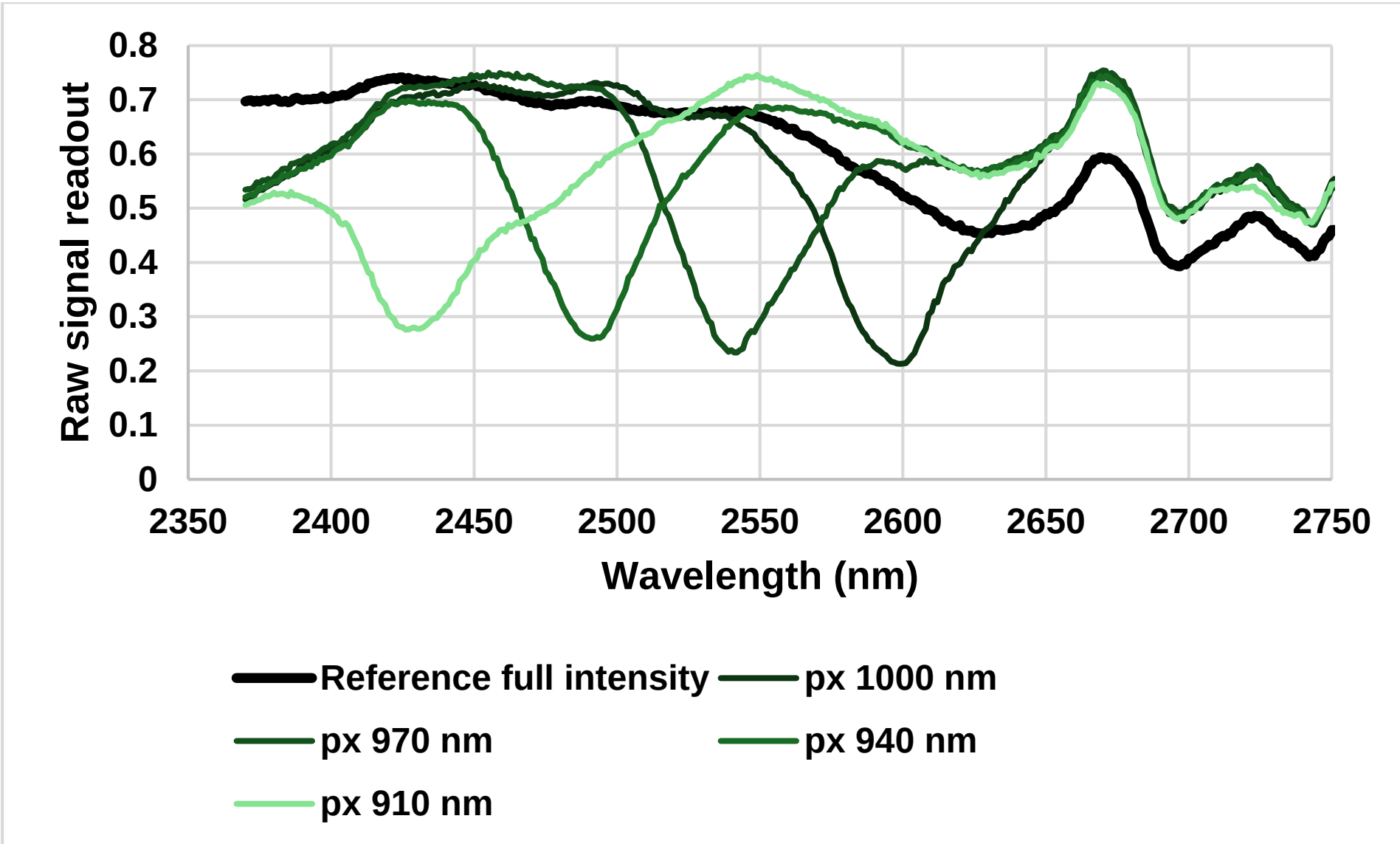

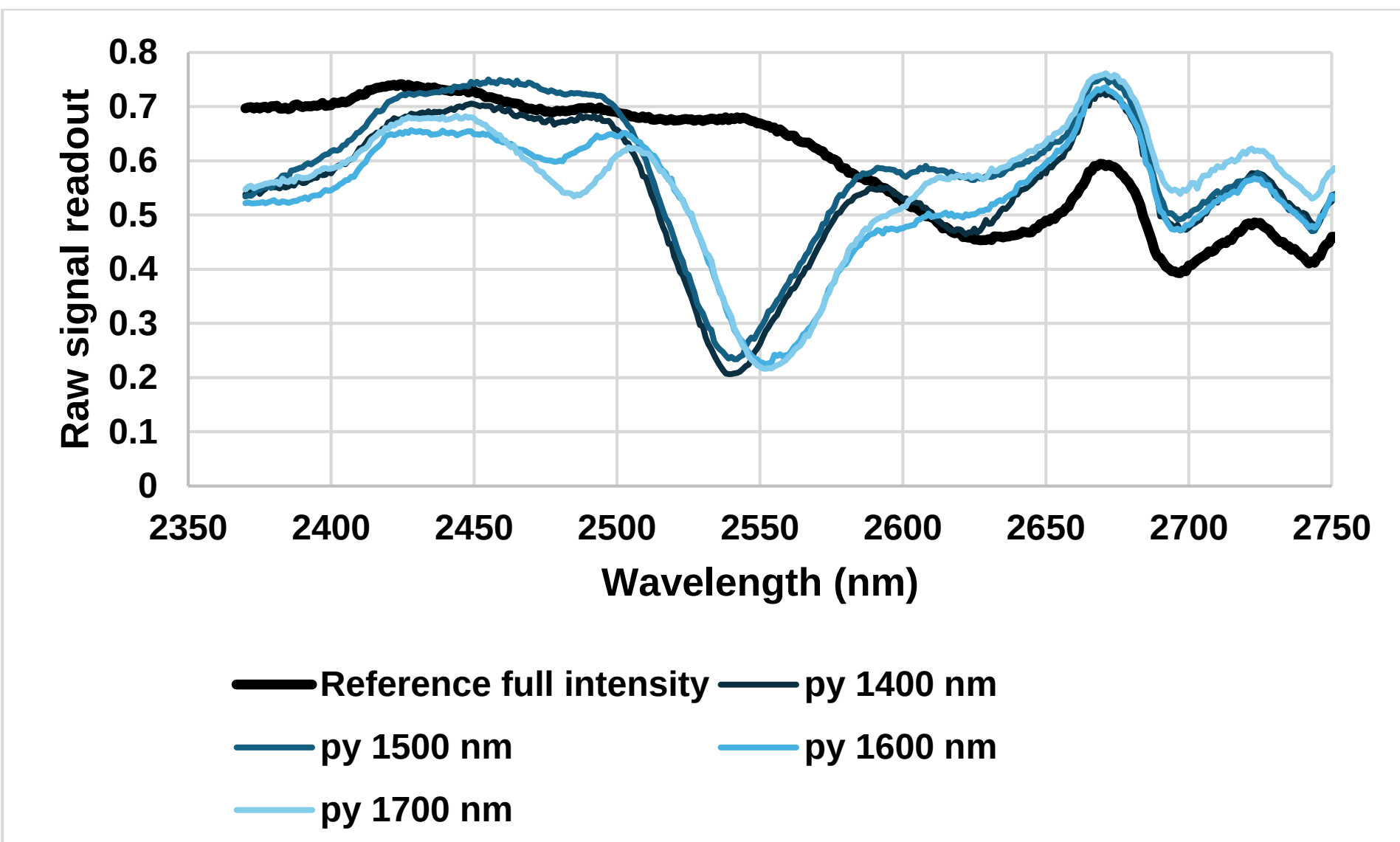

We divide the readout from the heaters by the full intensity to achieve the following curves. It is important to note that the full illumination is collected on a slightly larger scale (18 ^{o}C to 135 ^{o}C) and that there probably is some nonlinearity between power input at the pixels versus pixel readout at different wavelengths.

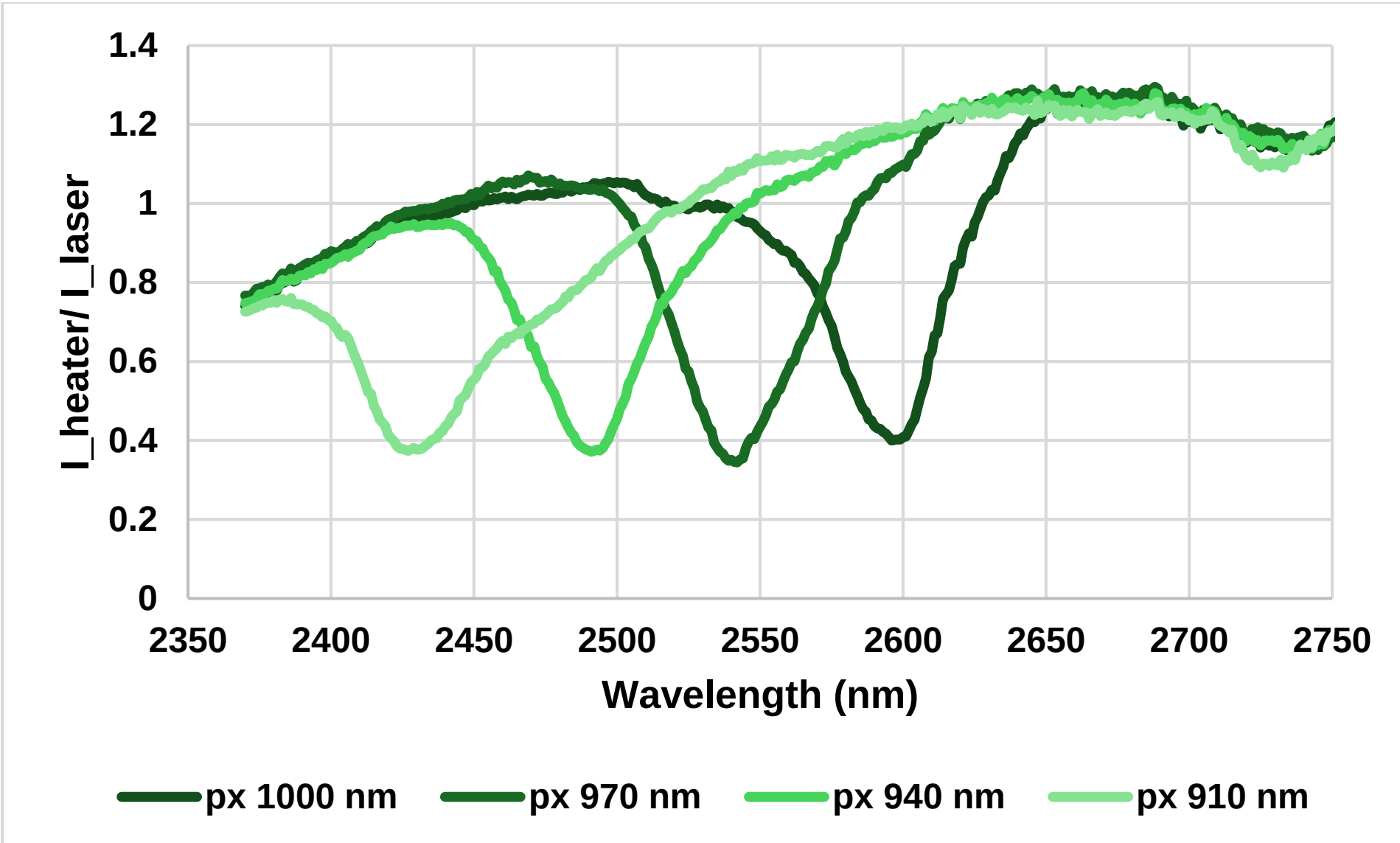

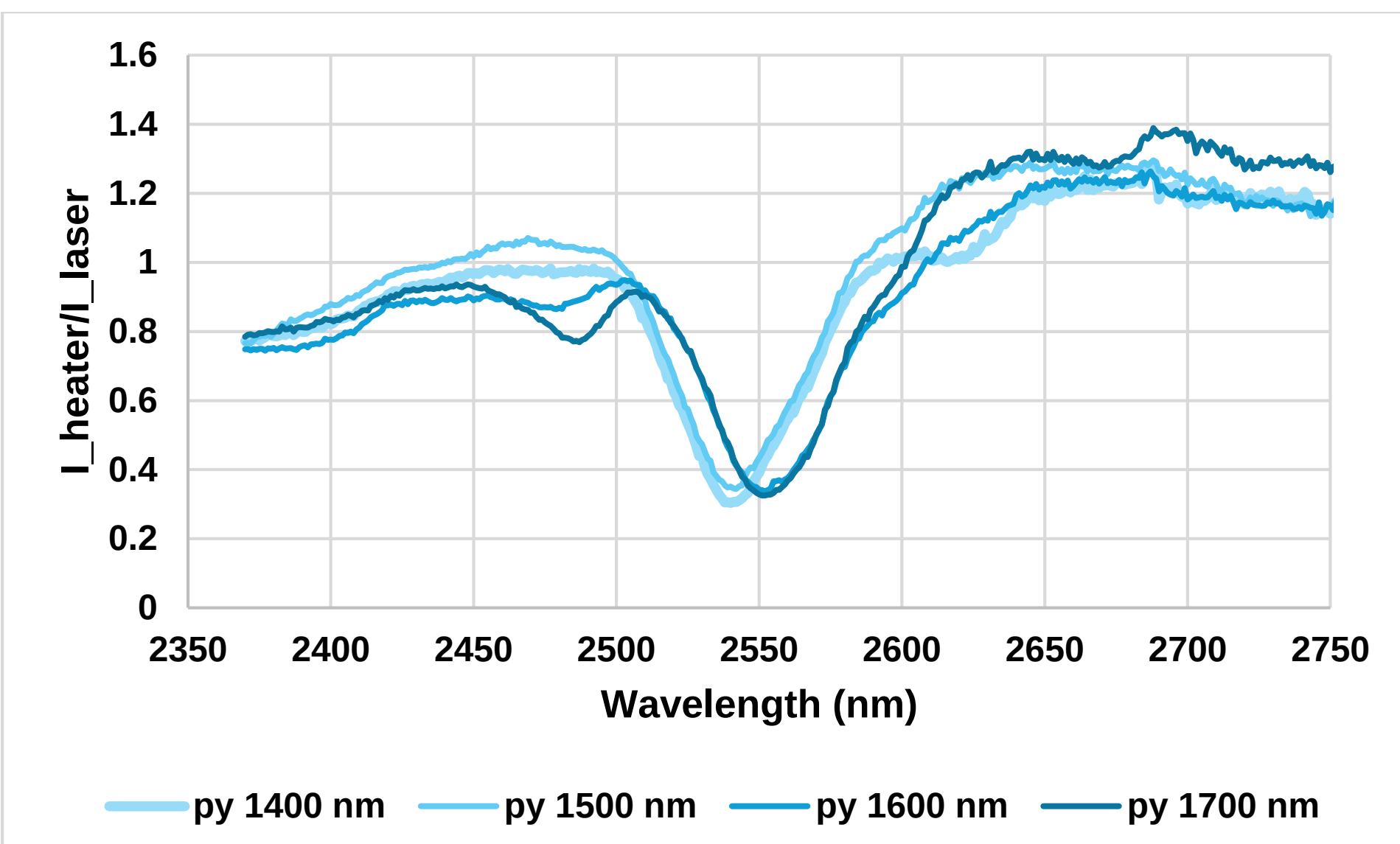

We normalize these graphs by the largest value within their set to yield the graphs in Figs. 3c and 3d in the main manuscript.

We agree that the silicon-air interface results in a loss of about 30% of the signal due to reflection, but this dissipation can be mitigated by anti-reflective coatings. We also agree that a quantitative power measurement is beneficial, but our results from the IR camera's image align with the expected behavior from simulations. Considering the lack of good power meters in the MWIR range, we believe our results strongly support the

existence of a resonance mode and the change of optical response upon phase transition of the GSST.

We have added in the main manuscript the following:

Device characterization. The devices were characterized using the setup shown in **Error! Reference source not found.**a. The chip was illuminated using a tunable Firefly M2 laser source (Firefly-IR-LP-A-BB-I) with pulse duration <10 ns and linewidth <10 cm^{-1}, which averaged out the Fabry-Perot interference of the silicon substrate. For spectral measurements, the wavelength was swept in 1-nm steps with a 0.1 s dwell time. The sweep was manually synchronized with a MATLAB routine that simultaneously collected images from a Telops IR SPARK M150 camera operating in radiometric temperature mode, scaled between 18 °C and 100 °C for better image sensitivity, except for the unimpeded laser illumination for which a setting of 18℃ to 135 ℃ to avoid pixel saturation. The lower threshold was chosen to be 18 ℃ to avoid stray signal from the laboratory environment. The images were collected on an 8-bit depth and normalized to a range of 0 to 1 (with 1 corresponding to 255 from the camera). The sample image was focused onto the camera, and the relative positions of the chip and camera were adjusted to achieve the desired magnification. Images were collected during the spectral sweep, and the pixel counts/intensity in the regions of interest were averaged. A separate spectral sweep was performed with the laser hitting the camera directly, without any sample on the pathway. The averaged values for the devices were divided by the values for the unimpeded laser sweep and normalized to the highest value within the data set to show relative trends.

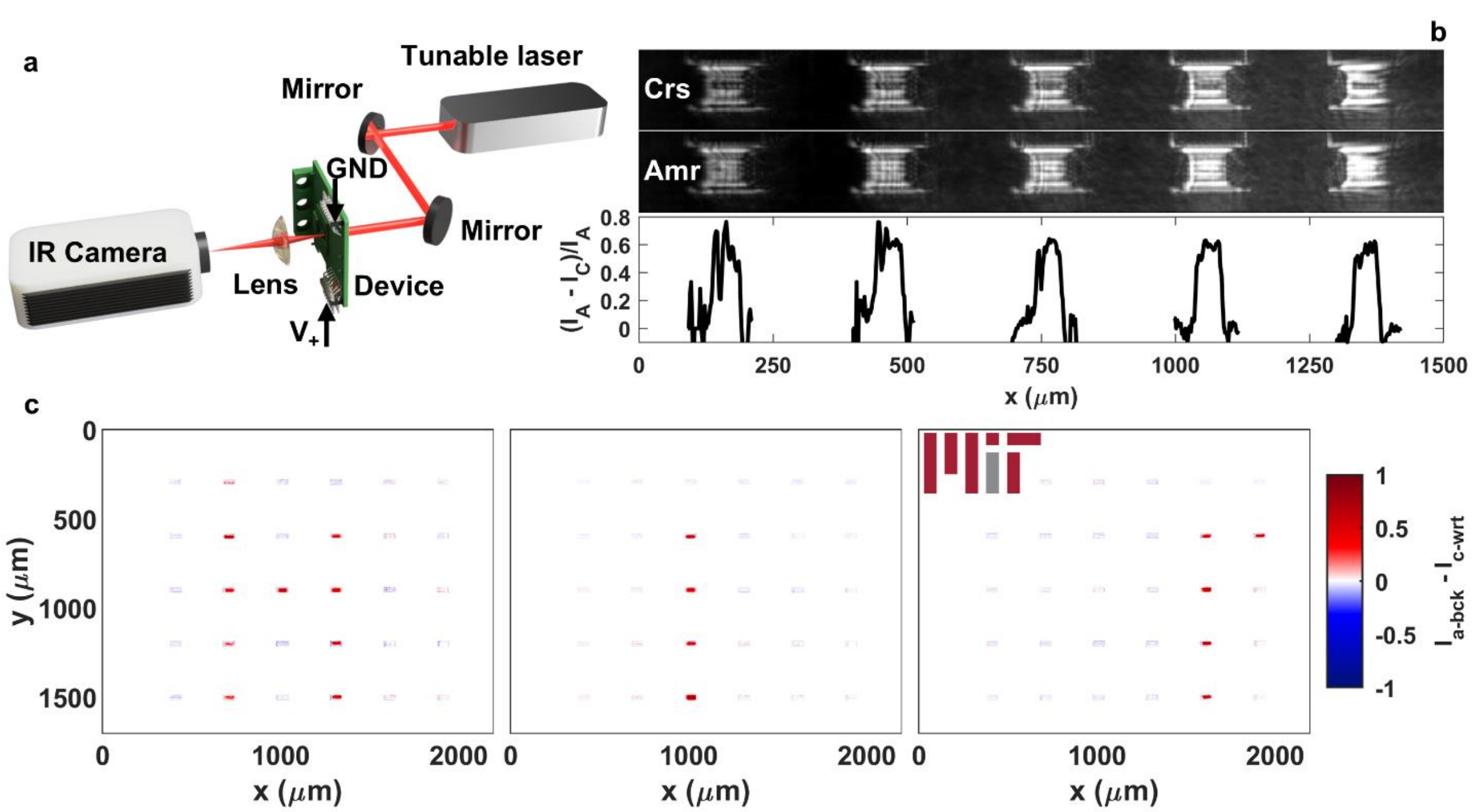

Furthermore, Fig. 4 has an unlabelled colorbar. What do negative values in it imply (and why one of the pixels in the right panel seems “broken”)? Are they coming from the interference fringes observed in the laser transmission image? This interference seems to be quite strong, which makes one wonder how the spectra from Fig.3 were achieved and whether the interference has some contribution to the observed signal magnitudes.

We thank the reviewer for noting this issue. The maps depicted show the difference in the optical transmission between the amorphous state (I_amr) and crystalline state (I_crs), where red is pure amorphous transmission and blue is pure crystalline transmission. The same definition is used in Fig. 3b.

To address the broken pixel, the image used for the “written” (or “crystalline”) state of “T” was the last in a set of images (the same one used in the supplementary movie 2). The “broken” pixel was amorphized as a test before our next experiment — the endurance tests. We mistakenly presented the image after the test as that for letter T. We have now replaced it with the correct image to represent the letter T.

The pixels were switched one by one, and the following images were collected to serve as the amorphous (background) state and crystalline (written) state.

Ideally, the same background image could have been used for all 3 letters, but the sample drifted slightly in position between the letter I and T. As a result, readout at the pixels also drifts, and we had to take separate backgrounds for the last letter to accommodate it.

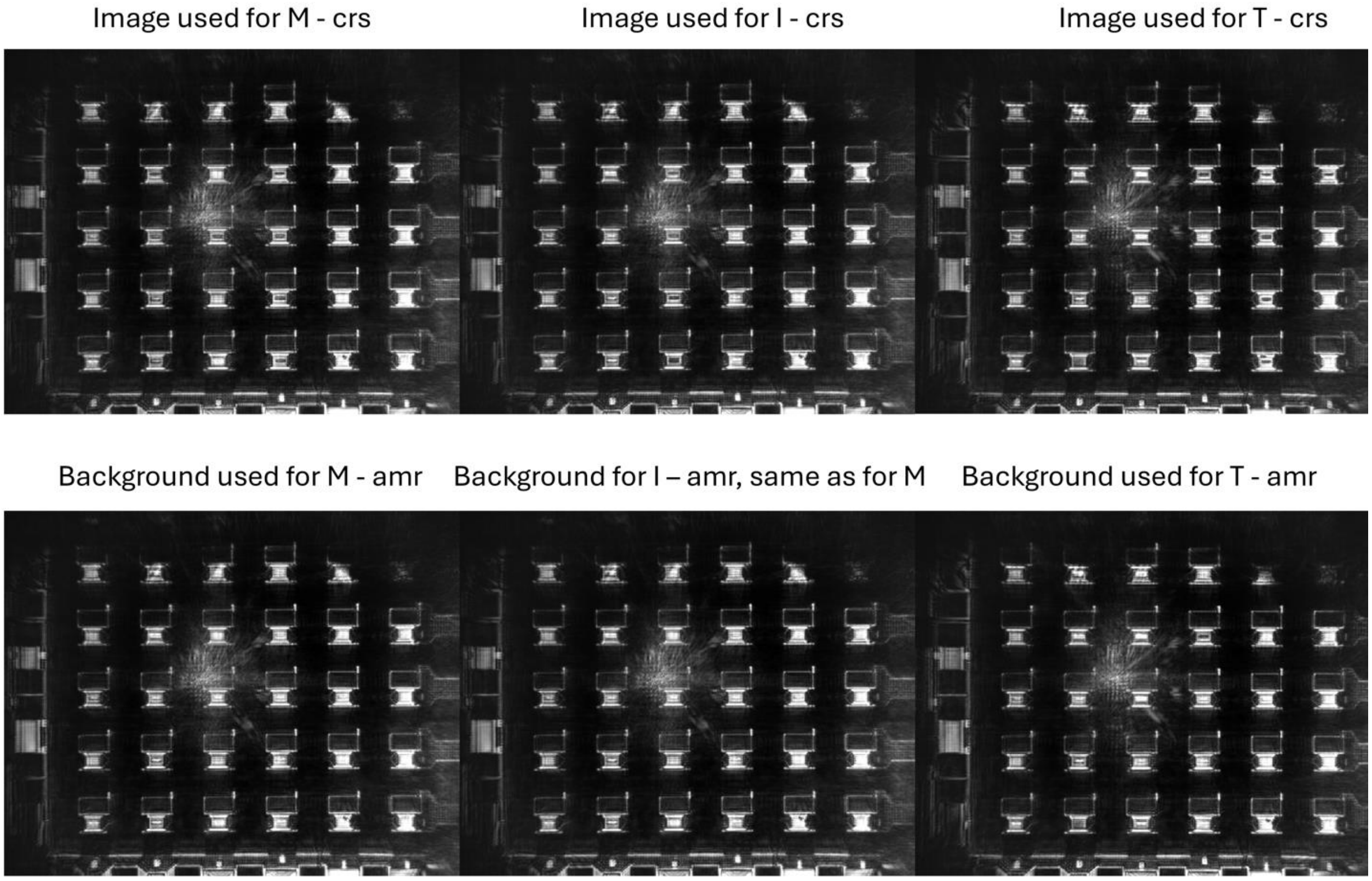

To highlight the areas that are switching, we mask out the regions outside the heater regions, and we get the following images. It is worth noting that treating the background state as the amorphous one at the chosen wavelength results in high transmission in the background state and low in the written state, similar to an “active-low” operation in electrical systems. This image processing could be avoided with the addition of metal apertures covering also the transition tapers of the microheaters and the other regions outside the metasurface, and with improved control of the illumination source.

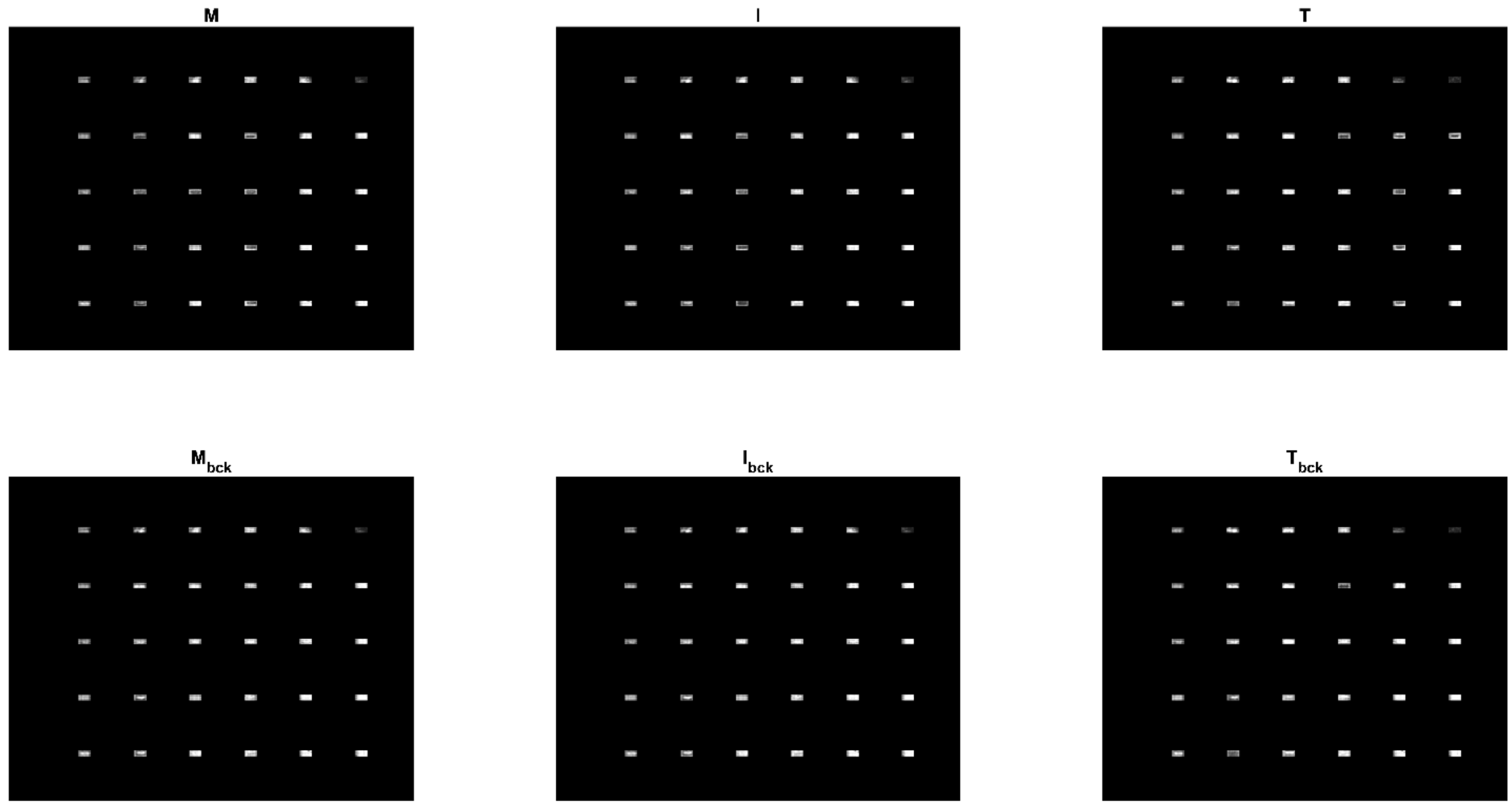

Negative data in the image is in part due to slight position drift of the sample during the data collection leading to slightly different illumination from image to image through the individual pixels and effectively resulting in noise, where in the written state a given pixel/microheater was actually receiving slightly more amount of light than in the image for the amorphous state. In a commercial product that encases the chip in a frame (e.g. a steel frame) and prevent vibration with respect to the light source, this type of noise is not expected to play a significant role.

We have updated the image in the manuscript with the corrected version and with the labeled color bar and corrected the caption to "**(d) 2D pixel transmission maps of the 6 × 6 array, generated by subtracting an image with the selected pixels programmed to display the letters "M," "I," and "T" in the crystalline (off) state from a baseline image with all the pixels in the amorphous (on) state.**"

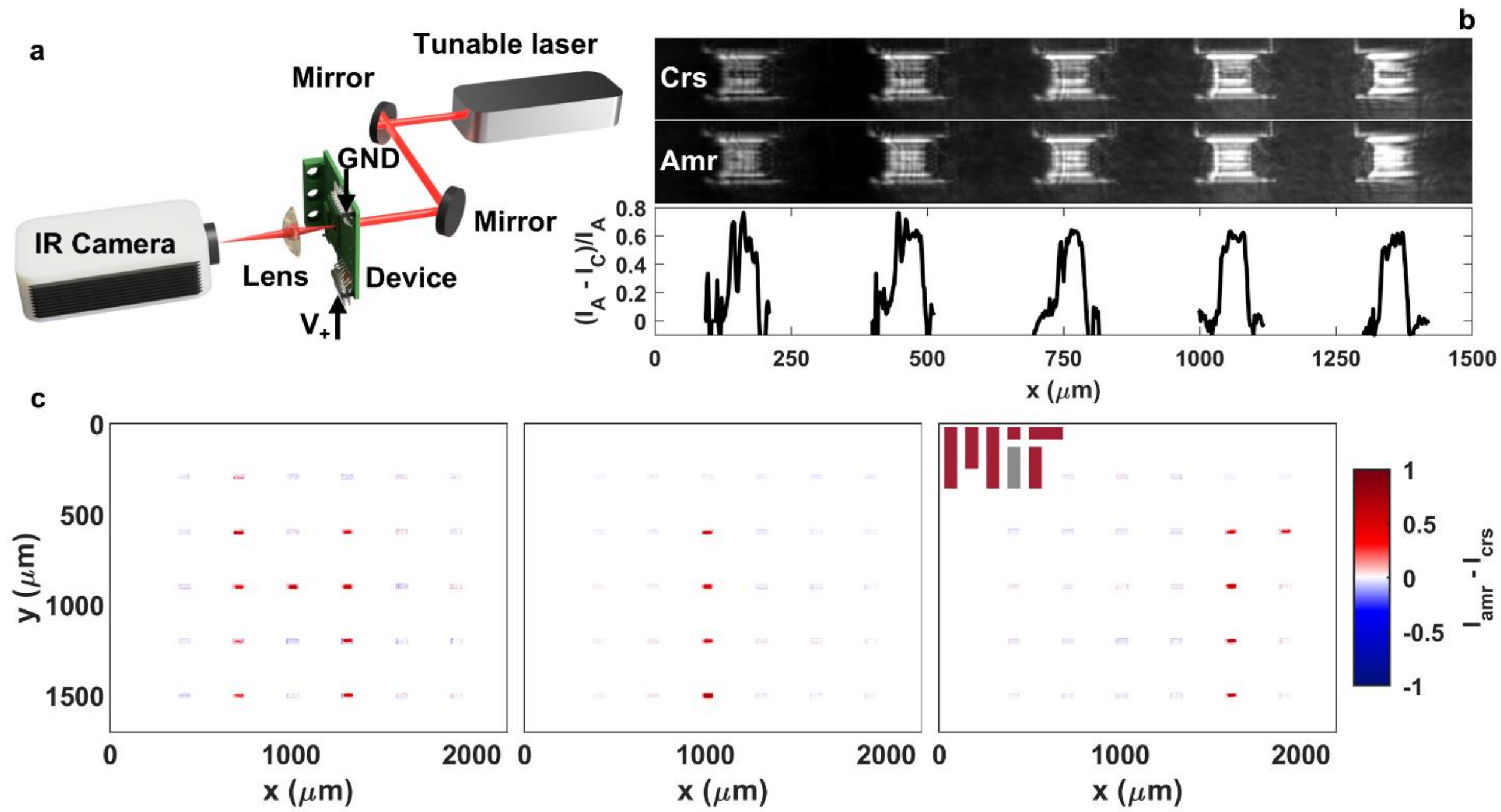

Furthermore, I would expect the spectra to be influenced by the FP interference in the silicon carrier substrate, particularly for parallel beam excitation configuration. Were they observed in the raw data and how they were processed?

We agree that the silicon substrate gives a FP interference and initially we expected to see them. It turned out that the pulsed laser we used is too wideband that averaged out the interference spectrum. We used a Firefly M2 (part number Firefly-IR-LP-A-BB-I, pulse width <10 ns and linewidth <10 cm^{-1}, according to the manufacturer). At 2500 nm wavelength, a 10 cm^{-1} difference is approximately 6.2 nm wavelength difference. For a 700 μm thick silicon substrate (a value expected for a foundry chip), the FP free spectral range is calculated as ≈1.4 nm. As such, the pulsed laser averaged out the spectrum of the FP fringes. While we do not have the exact linewidth of the laser, it is reasonable to expect that it is sufficiently larger than the FSR of the FP mode.

To add more information for the readers, we have added the following:

The chip was illuminated using a tunable Firefly M2 laser source, laser head part number Firefly-IR-LP-A-BB-I, pulse duration <10 ns and linewidth <10 cm^{-1}, which averaged out the Fabry-Perot interference of the silicon substrate.

3.Why does the device architecture used in the manuscript show an order of magnitude improvement in the endurance over the author's previous work (ref 33)? As the authors claim this is a significant advance, the design solutions that led to this improvement need to be elaborated. Furthermore, the way the data is presented currently makes one wonder if the pixel from 1e is just a lucky one that happened to be more robust. The authors need to provide more statistics from multiple pixels to support their claim.

From prior tests with doped silicon heaters, one primary reason for breaking the device is the electrical failure. In this device, we tried to mitigate such a failure by (1) using foundry fabricated tungsten plugs, and (2) isolating the electrodes from the high temperature area via the tapers. These practices ensured the heaters to survive an increased amount of heating events. The primary question is if we have a reason to expect fabrication imperfections on the last steps of the process where the PCM is added, patterned and encapsulated, as that will be the likely failure point for other pixels in this set.

Here are several of the improvements in the heater design vs the previous one:
1 – At AIM Photonics we had tungsten plugs to the silicon level, as opposed to the previous aluminum pads with only a few tens of nanometers separation from the silicon level through the adhesion layer. Furthermore, the tungsten plugs were separated from the high temperature region by 27 μm long tapers, combining physical distance with improved chemical and thermal stability, making this significantly more thermally resilient vs the old design. The old design was affected in part by the possibility of damage of the aluminum metal pads.

2 – In the previous work in Advanced Materials, we had metasurfaces with a period of 700 nm and the windows in the fishnet were around 450 nm, making the window area around 40% of the repeated unit cell. Meanwhile, the metasurface we have in this device has a window of 768 nm × 1345 nm with a period of 970 nm × 1600 nm, making the open window about 67% of the unit cell area. This increase, while not expected to be the primary contributor to the resilience improvement, is expected to contribute at least to some extent to allow better anchorage of the Al_2O_3 / SiN_x cladding and improve the endurance. This is based on the rationale that a smaller fraction of PCM in the unit cell will lead to decreased volume fraction change due to the phase change during operation and less stress. In other words, a smaller amount of PCM will make the system more resilient at the obvious cost of loss of tunability.

3 – A third feature that we hypothesize may have played a role in the endurance improvement vs the prior metasurface is the presence of the improved interface between the oxide and the silicon in the regions outside the medium doped N+ 40 × 60 μm region and separation of the high stress point for the capping. As figure 1 panel a illustrate, the nitride and oxide were etched only above the medium doped region, while leaving them unetched outside the heater and intended optical area. This hypothesis would require further validation (both computationally, such as multiphysics simulations, and experimentally), but it is based on a similar concept by Xia et al. (Xia, J., Wang, Z., Yang, R., Wang, T., Gong, J., Dong, Y., Li, Z., Yao, J., He, Q., Cheng, X. and Cheng, Z., 2024. Ultrahigh endurance and extinction ratio in programmable silicon photonics based on a phase change material with ITO heater. *Laser & Photonics Reviews*, *18*(4), p.2300722.)

As for the endurance test, we only conducted endurance test on one device due to the time-consuming nature of such experiment. This was primarily because of the long cool-down time required as a result of the lack of a heat sink. More specifically, each cycle took ≈30 s to minimize the accumulation of heat on the device. As such, it took 8 days for the endurance test on a single pixel.

We have added the following:

"Additionally, the demonstration of extended endurance enabled by the choice and optimization of capping materials and other advances, are readily scalable, suggesting promise in the fabrication of larger arrays for a range of optical functionality. Specifically, the usage of tungsten plugs from a foundry run, tapered regions avoiding current crowding and separating the electrodes from the high temperature areas, and increased landing area for the capping contributed to the increased switching resilience of the heaters themselves. Larger windows in the PCM structure also likely enable better adhesion of the capping and reduced delamination risk, albeit this was insufficient to completely prevent it."

4. The paper has a very detailed outlook, which compensates to some extent for the fact that the proof-of-concept device is lacking in many of the aspects (fill-factor, transparency band, etc.). I suggest complementing this outlook with discussion on thermal-diffusion-limited operation speed of the device, and its comparison to state-of-the art SLMs.

We thank the reviewer for the guidance. The speed of our devices is limited by crystallization. If we switched to a PCM with shorter crystallization kinetics, then we could reach into the regime where we are thermal diffusion limited. For a 2 μm buried oxide, the thermal time constant is around 5 μs and for a 90% settling state that would mean a speed of at least 80 kHz. In terms of switching speed, some of the fastest devices are MEMS that enable phase modulation via micromirror displacement on the scale of 200 kHz or more (reflective systems), and electro-optics SLMs on the scale of 1 GHz. Even so, our focus for PCMs in such a platform encompasses the wavelength range, with a focus on the mid-IR and far-IR, the non-volatility and the ability to operate in transmission.

We have added the following: "Realizing the full potential of fast PCM pixels will require pixel-level transistor integration for active-matrix addressing, an advance that our foundry-compatible platform is uniquely positioned to achieve. In this work, the switching speed is limited by the crystallization speed of GSST."

5. Do the authors envision their "recent work" (line 208) to be published in any format before this manuscript? If not, I suggest that the reference to unpublished data is removed.

Yes, the paper is under review in Advanced Materials.

Reviewer #2 (Remarks to the Author):

The manuscript describes an electrically tuneable phase change metasurface pixel array. The authors demonstrate the feasibility of electrically switching arrays of phase change pixels which, if scaled further, could be used to develop a useful metasurface. I think the technical achievements in this paper are worthy of publication in a Nature Comms. However, I believe the title of this work could be misinterpreted. When I read the title, I understood that each meta-atom was addressable. This assumption was then reinforced by the intro, which describe the importance of controlling individual meta-atoms to control the phase, polarisation, and amplitude of light. However, the authors have actually created an array of addressable fishnet-metasurfaces—not individually addressable meta-atoms within a metasurface. Addressable, metaatoms would be much more impactful because, in principle, the far field intensity and phase profile could be sculpted to produce a range of useful features in the far-field—such as holograms. Whereas the actual demonstration simply creates an array of absorber-transmitters.

Therefore, please can the authors increase the clarity of the title, and also early on in the manuscript explain that their aim for the present work is not to control individual meta-atoms.

We thank the reviewer for pointing out this lack of clarity. We have added the following:

” Here we present a scalable 2D electrically addressable active metasurface architecture that overcomes the scaling bottleneck, even if not reaching individual meta-atom control. The design combines BEOL-integrated PCMs with foundry-processed silicon microheater arrays addressed through multilayer backend metal interconnects, eliminating the need for complex fan-out wiring and avoiding interconnect congestion as array size increases.”

We have also changed the title to “2D Pixel-level Addressable Mid-infrared Metasurface Spatial Light Modulator”

Prior to publication, please can the authors also address the following points:
1. At EPCOS in Sept 2025, Prof Juejun Hu of also described excellent results for electrically addressable Sb2S3 (rather than GSST) pixels. Please can these results also be included in the present manuscript.

The work with Sb2S3 leverages atomic layer deposition and some other design choices that are not presented in the current manuscript. Furthermore, we are still collecting the data on those samples and we would like to publish it in a more comprehensive work once it is finalized.

2. Line 57, the authors discuss the advantages of PCMs over liquid crystals. I think they should also discuss scalability issues of LCs (e.g. anchoring effects and LC correlation lengths being similar to meta-atoms).

We thank the reviewer for the suggestions. We added the a more thorough discussion about liquid crystals:

Existing SLM platforms, such as liquid-crystal-on-silicon (LCoS) devices, suffer from strong absorption of liquid crystals in the mid-IR and relatively slow response times[21]. Furthermore, they can display anchoring and correlation effects that generate undulations and other defects on the scale of a few to tens of microns, competing with the desired optical output and generating potentially undesired higher order modes.

3. Why did the authors choose to use doped-Si heaters? Why not use ITO or some other IR-transparent conductor?

We thank the reviewer for asking this question. It is primarily due to the scalability of doped silicon—doped silicon heaters can be fabricated in any standard commercial foundry, while other materials such as ITO are not as easily available, especially for a research group to access a multi project wafer. The doped Si microheaters were fabricated by AIM Photonics, on top of which we conducted the back-end-of-the-line integration of PCMs(detailed steps see Methods). Using ITO or other materials would have required significant process development in a university cleanroom, adding a significant complexity to this research. The point of using a commercial foundry is to make easier the development of PCM-based optical devices. Moreover, for mid-IR applications, doped silicon can be an equally good option compared to ITO or other transparent conductors. In the main manuscript, we wrote "The device is fabricated through standard silicon photonic foundry processing combined with backend-of-line (BEOL) integration and employs multilayer backend metal interconnects to implement a crossbar addressing scheme" and "The result establishes a foundry-compatible route to high-density, large-area active metasurfaces with independently tunable pixels." The standardization is beneficial in scaling up and doped silicon is more easily accessible than other transparent conductors.

4. Why did the authors focus on GSST—please justify its use over other competing phase change materials.

We thank the reviewer for bringing this up. We chose GSST because it produces a good optical contrast thanks to its large contrast in refractive index and loss. Higher bandgap PCMs such as Sb_2S_3 and Sb_2Se_3 have lower refractive index change but also a lower loss, requires a significantly thicker layer compared to GSST in the mid-IR spectrum. As such, they are more suited for near-IR or visible applications. GST-225 can also be a candidate but it introduces quite higher losses compared to GSST. It is conceivable that with more optimization, GST-225 could also be used in the type of devices in this work.

We have added the following:

"This convergence of scalability, broadband operation, and nonvolatile programmability opens opportunities well beyond amplitude-only modulation. While we focused on GSST in this work due to its good balance of optical contrast and low loss in the mid-IR, the platform is PCM agnostic and other compositions can be chosen based on the required design specifications such as operational wavelength or switching time. With suitable metasurface designs, the same architecture can be adapted for"

5. Why does the device fail due to delamination? How can this effect be prevented?
We are delighted that the reviewer asked this. Phase change materials undergo a volume change between their amorphous and crystalline state (approximately 3% for GSST, a significant contribution, even with respect to simple thermal expansion, [Shalaginov, M.Y., An, S., Zhang, Y., Yang, F., Su, P., Liberman, V., Chou, J.B., Roberts, C.M., Kang, M., Rios, C. and Du, Q., 2021. Reconfigurable all-dielectric metalens with diffraction-limited performance. *Nature communications*, *12*(1), p.1225.]). Specifically, the amorphous phase will have a slightly larger specific volume compared to its crystalline counterpart. For the purpose of simplicity, I am ignoring the presence of multiple crystalline phases (such as the metastable cubic vs the stable hexagonal states). The amorphous state will result in higher pressure being applied to the protective layer (in this case the nitride capping) which, with time, will result in delamination. This can be prevented by restricting the amount of PCM in the structure or restricting the switching fraction, which unfortunately both limit the optical design, or could be addressed by altering the overall architecture of the device, to minimize the stress on the capping and distribute it more into the heater. Improved adhesion between the cladding and the heater would also limit delamination, and potentially also further alteration of the cladding geometry and composition could improve its mechanical resilience, such as a multilayer cladding, or a shallow etch into the cladding to redistribute the stress. Unfortunately, this is a relatively intensive thin film mechanics and fluid dynamics problem that is beyond the scope of this work. As mentioned in the main manuscript, the addition of wetting layers may limit the PCM from delaminating as that would improve the adhesion of the PCM to the heater.

We have added the following:

"The demonstrated improvement marks a significant advance in PCM metasurface durability, with further gains anticipated through the incorporation of wetting layers to enhance adhesion and suppress delamination, and optimization in the PCM and encapsulation geometry to minimize thermally induced and phase change induced stresses."

6. How does the cyclability depend on the set-reset voltage, current, and frequency?
The reviewer brought up an interesting point. High voltages will result in more energy being dissipated into the device, leading to higher temperatures and higher stresses and increased failure. Similarly, higher frequencies that result in switching the device before sufficient cool-down will result in accumulation of energy from cycle to cycle and crossing the temperature regime beyond which the thermally induced stresses in the device will lead to failure. This is part of the reason why we mention inversely designed

doped silicon microheaters to make the temperature profile more uniform, such that the maximum temperature reached in the device is reached over a larger area of the PCM and thus maximize switching volume and optical contrast while minimizing the risk of failure or accumulated damage to the interfaces and various regions of the device.

Another aspect that needs to be accounted for but which for these devices was significantly mitigated via the initial design is excessive current which could damage the electrodes and current carrying metal layers.

We have added the following:

“. Greater thermal uniformity is expected to improve the switching uniformity and usable active area of the device, without requiring excessive voltage application that can degrade the metasurface due to excessive temperatures.”

7. The cycleability in Fig 3(e) looks good but it would look even better if you added some statistics. Was this just the best cycling result? Or did all the devices perform to this standard?

We thank the reviewer for this critical comment. The result shows the only endurance tested device. Data collection for this type of devices is relatively time consuming in part because cool down needs to be allowed for the chip as the current implementation does not have a heat sink attached to the chip with the devices and it was simply kept mechanically in place by a 3D printed PLA structure. Specifically, a cycle is on the scale of 30 s to minimize overheating of the device, and it took 8 days of cycling for this test. From prior tests with doped silicon heaters, as long as they do not fail due to electrical failure (which we tried to mitigate via the usage of foundry fabricated tungsten plugs and isolating the electrodes from the high temperature area via the tapers to the central region), they do not fail to conduct electricity and heat up. The primary question is if we have a reason to expect fabrication imperfections on the last steps of the process where the PCM is added, patterned and encapsulated, as that will be the likely failure point for other pixels in this set. It is possible that this one device was a relatively good one, albeit we do not have reason to suspect it is particularly good or bad.

8. Fig 4d shows the M I T characters displayed on the image matrix. There was also a nice video uploaded showing these characters appearing. Although these images and videos clearly show the tunability of the pixel transmission, it seems there is still considerable room for improvement. I.e. the pixel uniformity for the ‘off’ and ‘on’ states seems to be an issue, and there are some rogue pixels. Moreover, the actual

transparent area of each pixel seems to vary considerably. Please can the origin of these issues be described in the main manuscript, and please suggest ways to resolve them in future iterations of the device.

Yes, we agree that the pixel uniformity can be improved. Also, we thank the reviewer for noting the rogue pixel. It was a mistake on the choice of the specific image used for the map generation and we have corrected the image in the main manuscript, even though all the relevant images are still part of the “Secondary Video 2”. We already note in the main manuscript that “We anticipate that incorporating designs such as inverse-designed microheaters with improved thermal uniformity40 can deliver similar or superior endurance in metasurface platforms.” And we have added the following “We anticipate that incorporating designs such as inverse-designed microheaters with improved thermal uniformity[40] can deliver similar or superior endurance in metasurface platforms. Greater thermal uniformity is expected to improve the switching uniformity and usable active area of the device. These changes can also account for the metal apertures needed to block stray optical signal which would have higher thermal conductivity.”

Furthermore, concerns over the thermal uniformity lead to us avoiding adding metal apertures above the doped silicon tapers, which would limit the streaks of light seen above and below the switching areas. With improved design, they can be addressed also.

9. To toggle between the amorphous and crystalline states voltage pulses of 25.5 V for 13 μs for amorphization and 18 V for 11 ms for crystallization were used. What current was used for these switching events? I believe the current density will be quite high.

We thank the reviewer for pointing out the technical detail. The electrical system available to us cannot measure the current drop on such a time scale, but we can estimate the current density.

From prior measurements on as-received devices at steady state, at 26 V one single heater dropped 0.0414 A. This would mean a current density through the 220 nm thick, 60 μm wide medium doped region (the region dedicated to the metasurface) is 3.14×10^9 A/m^2. At 18 V, the current dropped as 0.037 A and the current density (in the same region) was 2.80×10^9 A/m^2. In general, we aimed to keep current densities below 1×10^9 A/m^2 in the metal layers of the device to avoid damaging them. It is worth noting that on the microsecond and submicrosecond scale, the instantaneous current will be higher before the silicon has time to heat up to the point where we transition from the carrier concentration limited conducting regime to carrier scattering limited conducting regime. In other words, if we heat the silicon sufficiently, scattering will start to dominate and

limit the electrical conduction, acting as a self-limiter to how much power and current we can run through it.

10. On line 228 the authors suggest that the individual pixels could be addressed with transistor selectors. And that these selectors could be integrated on the BEOL by the foundry. We agree that this would be necessary for much higher density metasurface SLMs. However, we believe that this might be easier said than done. For the typical reset current densities (see point 9), how big would the AIM-foundry-defined transistor need to be to enable the GSST pixel to reset?

Unfortunately, AIM photonics specifically does not provide transistors as part of their fabrication capabilities, but other foundries that integrate transistors with photonic components could be used (e.g. Global Foundries). If we take a transistor with a drive current capacity of 1 mA/μm, then we would need 42 μm long transistors in place of the current diode to satisfy the steady state current drop. It is possible a 50% buffer needs to be provided to account for the initial higher current drop in the silicon, before it has time to heat up, so that may be a transistor on the scale of 60 μm long (comparable to the size of the current active region) so the limitations in scale would still originate in the metal layers that need to be designed to not exceed their respective maximum current densities. The reference for the order of scale of the drive current capacity is “Packan, P., Akbar, S., Armstrong, M., Bergstrom, D., Brazier, M., Deshpande, H., Dev, K., Ding, G., Ghani, T., Golonzka, O. and Han, W., 2009, December. High performance 32nm logic technology featuring 2 nd generation high-k+ metal gate transistors. In *2009 IEEE international electron devices meeting (IEDM)* (pp. 1-4). IEEE.” The 1 mA/μm value is relatively conservative, as higher currents/gate length are possible, with the following reference pertaining specifically to SOI lateral bipolar transistors. “Cai, J., Ning, T.H., Christopher, P.D., Yau, J.B., Chan, K.K., Yoon, J., Muralidhar, R. and Park, D.G., 2014. On the device design and drive-current capability of SOI lateral bipolar transistors. *IEEE Journal of the Electron Devices Society*, *2*(5), pp.105-113.”

We have added the following in SI:

One additional note on methods to increase the fill factor is the integration of transistors instead of the current diodes for microheater selection. From prior steady state measurements, at 26 V the system conducts 0.0414 A, and at 18 V, 0.037 A. This would mean a current density through the 220 nm thick, 60 μm wide medium doped region (the region dedicated to the metasurface) is 3.14×10^9 A/m^2 (or 0.314 MA/cm^2) and 2.80×10^9 A/m^2 (or 0.280 MA/cm^2), respectively. The peak current values are higher on the microsecond scale but last only a small fraction of the pulse, contributing a relatively small amount of energy. If we take a transistor with a drive current capacity of

1 mA/μm, then we would need 42 μm wide transistors in place of the current diode to satisfy the steady state current drop. To account for the peak current, a potential 50% buffer may be needed, placing the transistor size at ~60 μm, comparable to the size of the current active region, making the key limit still the BEOL interconnection and their current density thresholds. The width of the required transistor gate will scale approximately linearly with the width of the microheater region. All of this analysis is presuming CMOS only solutions, but other types of selectors may also be integrated in the future, such as ovonic ones which tolerate higher current densities.

11. The metasurface pixels are quite large. How would you expect the switching performance and cyclability performance of the device to scale as you reduce the pixel size down to a single meta-atom.

Reducing the metasurface to single meta-atoms may result in more reliable switching, as the stress from the PCM onto the surrounding regions (cladding, heater-cladding interface) will decrease and thus delamination will become less of an issue (starting to resemble phase change memory, such as 3D XPoint), but there will be problems in devices design and in optical performance or in electrical system implementation. If we maintain a similar scale for the heaters, we basically have a PCM scattering point with potentially fewer uses. If we scale down also the heater so the overall PCM density in the device remains the same, the wiring will be close enough to the optical part of the system that we need to take it into account, which will introduce a new level of complexity. This will be needed for individual meta-atom control but this level of control will require more development. In this situation, we can foresee that transparent electrical conductors such as ITO can be leveraged, but the process will require a partnership with a foundry where PCMs and ITO backend interconnects are both provided.

Reviewer #3 (Remarks to the Author):

I co-reviewed this manuscript with one of the reviewers who provided the listed reports. This is part of the Nature Communications initiative to facilitate training in peer review and to provide appropriate recognition for Early Career Researchers who co-review manuscripts.

We thank the reviewer for their contributions and input.

Reviewer #1 (Remarks to the Author):

In the revised manuscript, the authors satisfactorily addressed most of my comments. I recommend the paper for publication, but still suggest to add the discussion on the last point that was not properly covered in the revision.

This is connected to my questions 3 and 4; what I meant by thermal diffusion-limited operation speed is exactly what authors mentioned in their response to the question on the device cyclability (30 s dwell time was needed to address the issues of the device overheating). I suggest the authors mention what is the total dissipated power in the device operation regime and at which operation speed the issue can be addressed with a heat sink adequate for the chosen device format.

We thank the reviewer for the supportive statements and for the advice. We added the following:

"The averaged values for the devices were divided by the values for the unimpeded laser sweep and normalized to the highest value within the data set to show relative trends. During the endurance testing, the average time per cycle was around 30 seconds to allow sufficient cooldown for the sample in absence of any heat sink, considering that the chip was suspended in a 3D printed polylactic acid enclosure with a small support structure to not impede the optical signal."

"Realizing the full potential of fast PCM pixels will require pixel-level transistor integration for active-matrix addressing, an advance that our foundry-compatible platform is uniquely positioned to achieve. In this work, the switching speed is limited by the crystallization speed of GSST. During the endurance testing, to prevent overheating of the device, a 30 s wait time was used for the device to cool down via air convection. The chip was suspended in a plastic enclosure, with only minimal contact area between the chip and the enclosure. On average, the heat dissipated during a cycle for 18 V pulses passing 35 mA is around 7 mJ, almost all the energy being just the crystallization part, as the time for crystallization is 3 orders of magnitude larger than for amorphization. The addition of a heat sink would vastly increase the heat extraction and allow increasing the switching speed, with commercial heat sinks for similar sizes of the chip being expected to remove heat on the order of 1 W."

Reviewer #4 (Remarks to the Author):

The manuscript reports the realization, numerical, and experimental investigation of a pixel-level electrically addressable transmissive metasurface operating in the mid-infrared. The device is based on phase-change materials, integrates microheater-driven switching, and relies on a crossbar addressing architecture implemented in a silicon photonics platform. The device demonstrates programmable transmission patterns using a 6×6 pixel array fabricated in a foundry-compatible BEOL process, aiming toward scalable electrically programmable meta-optical devices. The work combines metasurface design, PCM electro-thermal switching, and integrated addressing circuitry to realize a proof-of-concept mid-IR programmable optical modulator.

The technical content is sound, the writing is clear, and the topic is timely and aligned with the journal's audience.

In my view, the authors have satisfactorily addressed all but one of the concerns raised during the previous review round, and the manuscript has improved accordingly. However, one concern raised in the previous review round appears only partially addressed, namely, regarding the quantitative interpretation of the optical measurements. While the authors clarified that the transmission values are derived from camera intensity measurements, the data remain non-absolute (see also question 2a below).

We thank the reviewer for the feedback.

I have however identified a few elements that would benefit from further elaboration prior to endorsing the publication of this article.

1) The manuscript advances several claims of novelty that appear somewhat overstated when compared with the cited literature.

a. First, the authors claim the "first transmissive mid-IR spatial light modulator (SLM)" based on a metasurface. While the mid-IR transmissive implementation is indeed distinctive, prior works have already demonstrated transmissive electrically addressable PCM metasurfaces and SLM-like devices in the near-IR (e.g., Fang et al., 2024; Popescu et al., 2024). Thus, the novelty here seems primarily the spectral range (mid-IR) rather than the transmissive PCM metasurface SLM concept itself.

We thank the reviewer for the comment. Yes, one distinction with respect to the prior works is the operation in the mid-IR. The work by Fang et al. 2024 shows transmission results up to 1600 nm and our prior work (Popescu et al. 2024) showed the operation on

a single pixel at a time with the images being collected in the near-IR. We have emphasized in the manuscript that this is a mid-IR operation. We have mentioned that this device operates in the mid-IR several times:

“In sum, the platform demonstrated here lays the groundwork for a new class of scalable, pixel-addressable active metasurfaces **across the mid-IR** and other spectral regimes”

“Demonstration of 2D electrically addressable transmissive **mid-IR** SLM operation”

“integrated metasurfaces and apply it to realize the first transmissive **mid-infrared (mid-IR)** amplitude-only spatial light modulator (SLM). The device is fabricated through standard silicon photonic foundry”

“To our knowledge, it represents the first report of a transmissive **mid-IR** SLM”

b. Second, the manuscript claims the “first 2D electrically addressable active metasurface with a scalable architecture.” However, earlier studies have reported 2D electrically programmable metasurface arrays with pixel-level control, even if implemented with different wiring schemes (e.g., Li et al., Nat. Commun. 2020 and related works). The true distinction of the present work appears to lie in the crossbar addressing scheme with integrated selectors enabling scalability, rather than 2D electrical addressability per se.

We thank the reviewer for the comment and we would like to clarify and strengthen the manuscript. The work by Fang et al. 2024 as well as the work by Li et al. 2020 showed a fan out designs which are harder to scale up, as for n^2 devices you would need approximately n^2 electrodes. This cross-bar array translates a n^2 array into 2*n inputs, similar to a lot of other devices which makes this platform scalable. In the prior submission we stated “The design combines BEOL-integrated PCMs with foundry-processed silicon microheater arrays addressed through **multilayer backend metal interconnects**, **eliminating the need for complex fan-out** wiring and **avoiding** interconnect **congestion as** array **size increases**.” And also “While one-dimensional electrical addressing can be realized with straightforward fan-out architectures[10–17], **extending this approach to two dimensions** is only feasible for **very small arrays** containing a **handful of independently controlled pixels** (Table 1)[18–20]” and “first 2D electrically addressable active metasurface with **a scalable architecture**”.

We have added to the manuscript the following:

“the first realization of a 2D electrically addressable active metasurface with a scalable architecture (as opposed to fan out architecture), and the first report of a PCM-based metasurface fabricated using a standard silicon foundry process with full BEOL integration”

c. Finally, the paper suggests that it is the first PCM-based metasurface fabricated using a standard silicon photonics foundry with full BEOL integration. However, foundry-compatible BEOL PCM integration has already been demonstrated for photonic devices (e.g., 10.1021/acsnano.4c00340).

We thank the reviewer for this comment. It is true that there has been prior integration of PCMs in BEOL of foundry fabricated photonic devices such as in the work by Adya et al. 2024 (https://doi.org/10.1364/OE.526141), in this example the Advanced Micro Foundry. In this example, Adya et al. used evanescent coupling of the mode into the GST PCM, with the photonic device being fabricated in the foundry. The work by Fang et al. in 10.1021/acsnano.4c00340 was primarily done in a research cleanroom, with the metasurface being defined after the ion implantation and before metallization, with the ion implantation being the only process outsourced to an external vendor.

We would like to reiterate that our devices are based on metasurfaces that are integrated in foundry fabricated devices, specifically AIM Photonics in our case, being separated from photonic devices which require much smaller volumes of PCM to be switched at a time and where the heater areas are also significantly smaller. To our knowledge, we are not aware of another demonstration of PCM metasurfaces being integrated into BEOL of standard silicon foundry fabricated devices.

d. There is at least one work investigating Pixel-level addressable SLMs for the mid-IR, 10.1364/AO.54.010698, albeit not leading to a practical realization.

We thank the reviewer for bringing up this example. In the work by Hendrickson et al., there are two designs shown with GST in between two layers, in one case two n-type Si layers and in the other one silver/ GST/ n-type doped Si. Depositing silicon on top of a PCM after the PCM deposition requires fabrication considerations. Specifically, it cannot be polysilicon as this type of silicon would require raising the temperature of the device to values at which the PCM risks degrading, such as starting to oxidize (if in oxygen environment), or volatilize due to the loss of the chalcogenide (depending on the chalcogenide used). Even if the PCM does not allow volatilization and there is no reactive gas to alter its composition, n-type silicon would likely still require temperatures close to the melting point of a lot of PCMs, including GST 225 (~ 600 C). The silicon could be sputtered, along with a dopant, but its electric conductivity will suffer without an

activation. Furthermore, the authors mention that "the PCM was sandwiched between transparent conductive N-doped SI or indium tin oxide contact layers in a simple planar structure". A simple thin film planar structure will likely lead to delamination of the PCM film if the thin film is large enough in the planar dimensions. Our work makes use of the geometry of the metasurface both for optical purposes (as a guided mode resonance) and also to prevent delamination.

We have altered the manuscript as follows:

"While there were some prior forays into designs of mid-IR PCM SLMs , transmissive mid-IR SLMs remain relatively unexplored, despite their advantages for compact optical layouts, cascaded optical processing, and integration with transmissive sensing or imaging systems."

To avoid priority conflicts, the authors should narrow their claims to emphasize the combination of selector-enabled crossbar addressing, transmissive mid-IR metasurface operation, and realization within a foundry-compatible BEOL platform, rather than broadly claiming first demonstrations of electrical addressability or BEOL PCM integration. Moreover, it would be useful to expand the literature review to include numerical investigations, rather than limiting it to practical implementations.

A set of prior works that present designs of PCM mid-IR modulators

Jiang, X., Zhang, Z., Ma, H., Du, T., Luo, M., Liu, D. and Yang, J., 2022. Tunable mid-infrared selective emitter based on inverse design metasurface for infrared stealth with thermal management. Optics express, 30(11), pp.18250-18263.

He, J., Shi, Z., Ye, S., Li, M. and Dong, J., 2022. Mid-infrared reconfigurable all-dielectric metasurface based on Ge2Sb2Se4Te1 phase-change material. Optics Express, 30(19), pp.34809-34823.

Liu, Y., Mao, R., Zhang, P., Li, W., Wang, M., Zhang, L., Gong, Y., Zhou, P., Liang, D. and Deng, L., 2022. Numerical study of mid-infrared tunable metalenses based on Ge2Sb2Te5 phase-change material. Journal of Physics D: Applied Physics, 55(24), p.244003.

We have added the following to the manuscript:

"While there were some prior forays into designs of mid-IR PCM SLMs , transmissive mid-IR SLMs remain relatively unexplored, despite their advantages for compact optical

layouts, cascaded optical processing, and integration with transmissive sensing or imaging systems.*”*

2) Several technical aspects of the manuscript would benefit from clarification or additional analysis. These points, taken individually, do not strongly affect the authors' key claims; however, addressing them would improve the quality of the work and strengthen the manuscript.

a. *The reported transmission maps and contrast metrics appear to be derived from camera intensity values rather than calibrated optical transmission. Since the data are normalized to detector response rather than referenced to incident optical power, the reported contrast and uniformity cannot be interpreted as absolute transmission or efficiency. The authors may therefore consider either pursuing a calibrated transmission measurement or reference normalization; or, alternatively, explicitly framing the current results as relative estimates limited by the experimental setup.*

   We thank the reviewer for the comment. The infrared camera used does not provide information about the power received per pixel and it is not feasible in the current setup to incorporate also an absolute power measurement system. We have further emphasized in the manuscript that these are relative estimates due to the limitations of the experimental setup.

   “in both amorphous and crystalline states. Due to limitations of the experimental setup, without the possibility to obtain absolute power measurements, these results are only relative estimates based on image analysis of the images collected by the Telops camera. The experimental spectra exhibit a systematic blue shift of the GMR wavelengths relative to design values and reduced extinction ratios, which we attribute to slight deviations between the actual and assumed refractive indices of the PCM and doped-Si layers.”

b. *The manuscript acknowledges parasitic transmission through doped-Si regions outside the active metasurface. This background signal likely contributes to the measured “on” state and may affect the reported contrast. The impact on the true optical extinction ratio and device efficiency should be quantified, or at least discussed.*

Quantifying the stray signal through the doped Si transition wings is not straightforward, but whatever contribution it has, this value would be added to both the on and off state, so in terms of contrast, accounting for the additional stray signal or altering the device to remove this stray signal would improve the contrast value. For a contrast of $\frac{I_{on}}{I_{off}} > \frac{I_{on}+I_{stray}}{I_{off}+I_{stray}}$ for all of them being positive values and $I_{on} > I_{off}$. Furthermore, while the usage of a camera for signal analysis does not provide us with absolute power analysis, we are able to isolate in the image the region that we process, meaning that we do limit to some extent the stray signal contribution to the analysis. It is true that using this as an actual device would suffer in performance, and further development on this platform would target blocking all the stray signal that does not get processed by the switching PCM, but by using a region of interest in the image analysis, this issue is partially limited at the analysis level. We added the following:

"The resonance wavelength exhibits a much stronger dependence on px than on py, in excellent agreement with the simulation trends in Fig. 2c and Fig. 2d. While there is some contribution from the stray light in the system (such as from the doped Si transition regions which protect the electrodes and were not covered with an absorptive aperture) accounting for this stray signal or removing it from the device would increase the contrast between the two states."

c. Key performance metrics such as absolute transmission, insertion loss, and optical throughput are not reported, making it difficult to assess the practical performance of the device as a spatial light modulator (e.g. an extinction ratio of 5.1 dB has a different practical impact, whether the transmission is absolute or normalized).

While it is not feasible to accurately measure the system power transmission (especially as there are many regions that will have varying transmission values such as – regions with the switching PCM, doped Si transition wings, other regions on the chips with dummies from the metal layers above) the estimated power transmitted can be estimated from other factors as well. Most of the power loss for these devices is estimated to come from the air-silicon interface (in the z-direction through the device) and from the blocked/rejected because it is not in the active region of the device. Air-silicon interface should give about 70% transmission and the RCWA simulations in Fig 2 show approximately 60% maximum transmission after accounting for the air-silicon backside, and the medium doped region (intended switching region, ignoring non-uniformities in the PCM switching) is 40 μm x 60 μm in a period of 300 μm x 300 μm, making it about 26% of the area of a unit cell. This combined with the maximum transmission from RCWA would place the maximum transmission at 16% (ignoring also stray signal). This calculation presumes uniform illumination but one potential way of

avoiding that is using a commercial micro-lens array of equal period to concentrate the signal through the microheaters. Of course, future optimization will drive up the fill factor of the heater region and switching PCM region, hence improving that factor. Furthermore, an optimized backside coating of, for instance, Silicon Nitride / Silicon Oxide/ Magnesium Fluoride or any other anti-reflective coating stack could reduce significantly the losses from the air-silicon backside interface. As this work has highlighted, there are many layers of consideration: heater design, electrical interconnects, selector design, PCM metasurface, protective cladding, etc, but more aspects can be optimized. One potential action we could have taken was a backside $\lambda/4$ SiO2 backside coating. Even then, getting the power signal from one single heater is challenging in our current experimental setup. While we agree it would add more context to this work, its absence does not take away from the demonstration that this is a non-volatile mid-IR SLM based on PCM metasurfaces.

d. In the demonstrated passive-matrix crossbar scheme, pixel programming appears intrinsically sequential, implying a rewrite time that scales as n2 for an n×n array. Given the reported switching times (~13 μs amorphization and ~11 ms crystallization), full-frame rewriting would become impractically slow for large arrays. Conversely (as a gedankenexperiment), fully parallel programming would require extremely large instantaneous power and heat dissipation, potentially causing thermal cross-talk and compromising amorphization due to reduced quenching rates. A discussion of the scaling trade-offs concerning write/erase time and energy would strengthen the manuscript.

In principle, the device could be switched line-wise (one row or column at a time) using appropriate electronic controls, as long as the electrical interconnects are designed to allow all that current simultaneously. The cross-talk originating from in-plane heat diffusion is not a significant concern for this design, partially because the heat generation occurs in a structure 0.22 μm thick (the doped silicon), the heat diffusion of interest occurs in the adjacent structures (with thicknesses of 0.7 μm SiNx, 0.22 μm GSST, 0.22 μm Si, 3 μm SiO2, ~4.14 μm before the substrate), while the in-plane dimensions of interest are on a larger scale, with 27 μm being the length of the of one of the transition tapers/wings to the electrodes. It is true that cool down needs to be allowed (and a proper heat sink connected) in order to speed up the repeated switching of these devices, but for one given frame change, the in-plane cross-talk is not expected to contribute significantly for these devices. If they were to be redesigned with a larger active optical area, then cross-talk concerns are valid, which of course becomes an

optimization problem. We have performed COMSOL simulations with period boundary conditions, presuming all the heaters undergo the same switching process.

Furthermore, as far as amorphization is concerned, the amount of heat injected is several order of magnitude less than during crystallization because the heating occurs on the scale of 10 µs. This means that the cooldown to amorphize should not be impacted, even if all the heaters were to operate simultaneously. Meanwhile, for crystallization, the maximum temperature desired is significantly below the melting point, and any cross-talk to adjacent heaters will result in temperatures below that. If the injected heat is sufficient to reach the Tc only at the heater, then the cross-talk, while on a longer time scale, will not be sufficient to trigger a change in an adjacent heater.

After running additional COMSOL Multiphysics simulations, while turning on multiple heaters adjacent to each other, the areas that are heated up are still confined to nearby the microheater, in part due to the insulative properties of the buried oxide and the time scale of the heat injection. Furthermore, while not implanted in the current work, further thermal isolation can be integrated by etching down the areas around the microheater, specifically etching the nitride + oxide from the foundry and then perform DRIE into the silicon. This will lead to further heat confinement and prevent thermal cross-talk further.

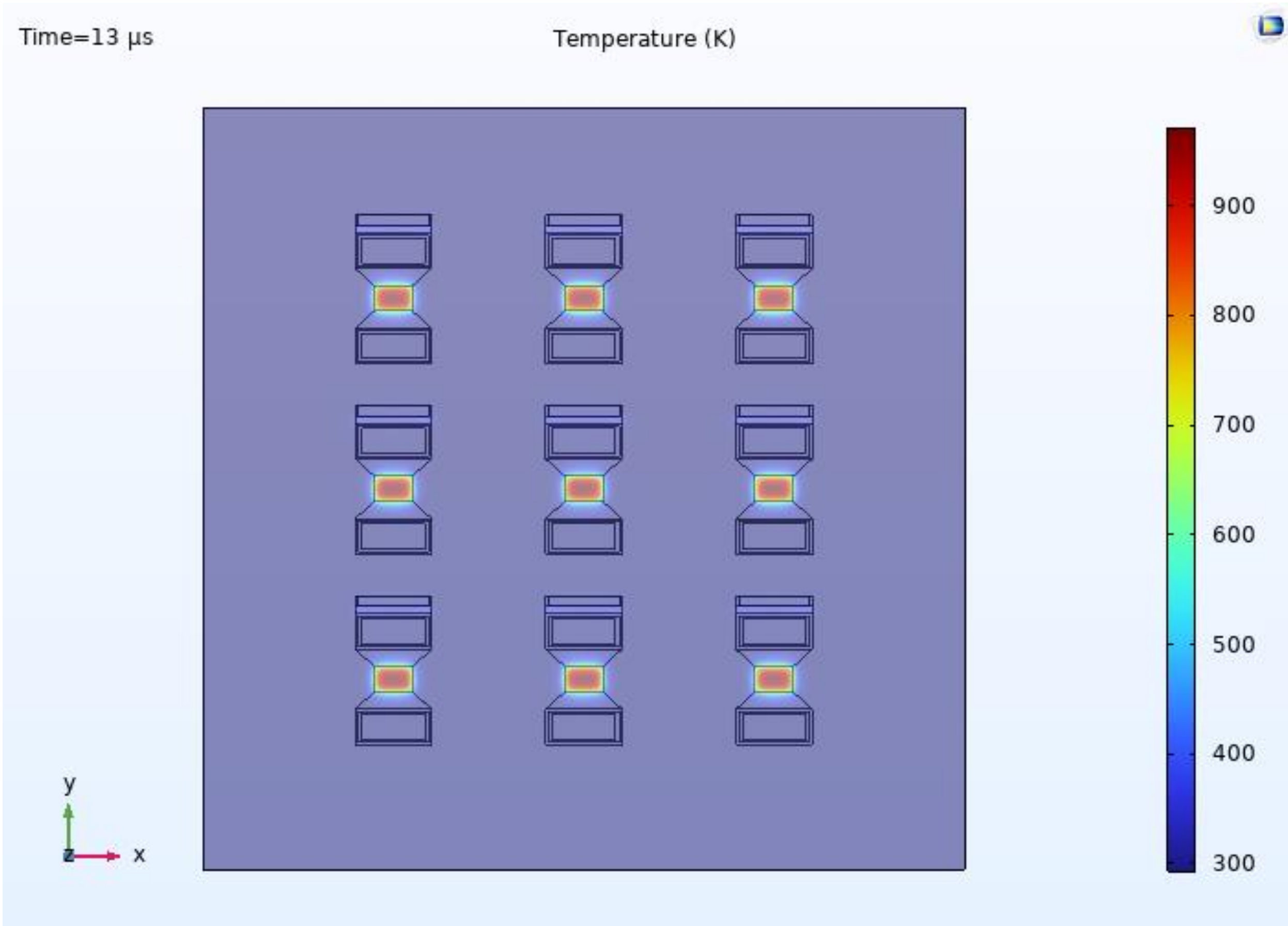
Time=13 µs
Temperature (K)
900
800
700
600
500
400
300
y
z
x

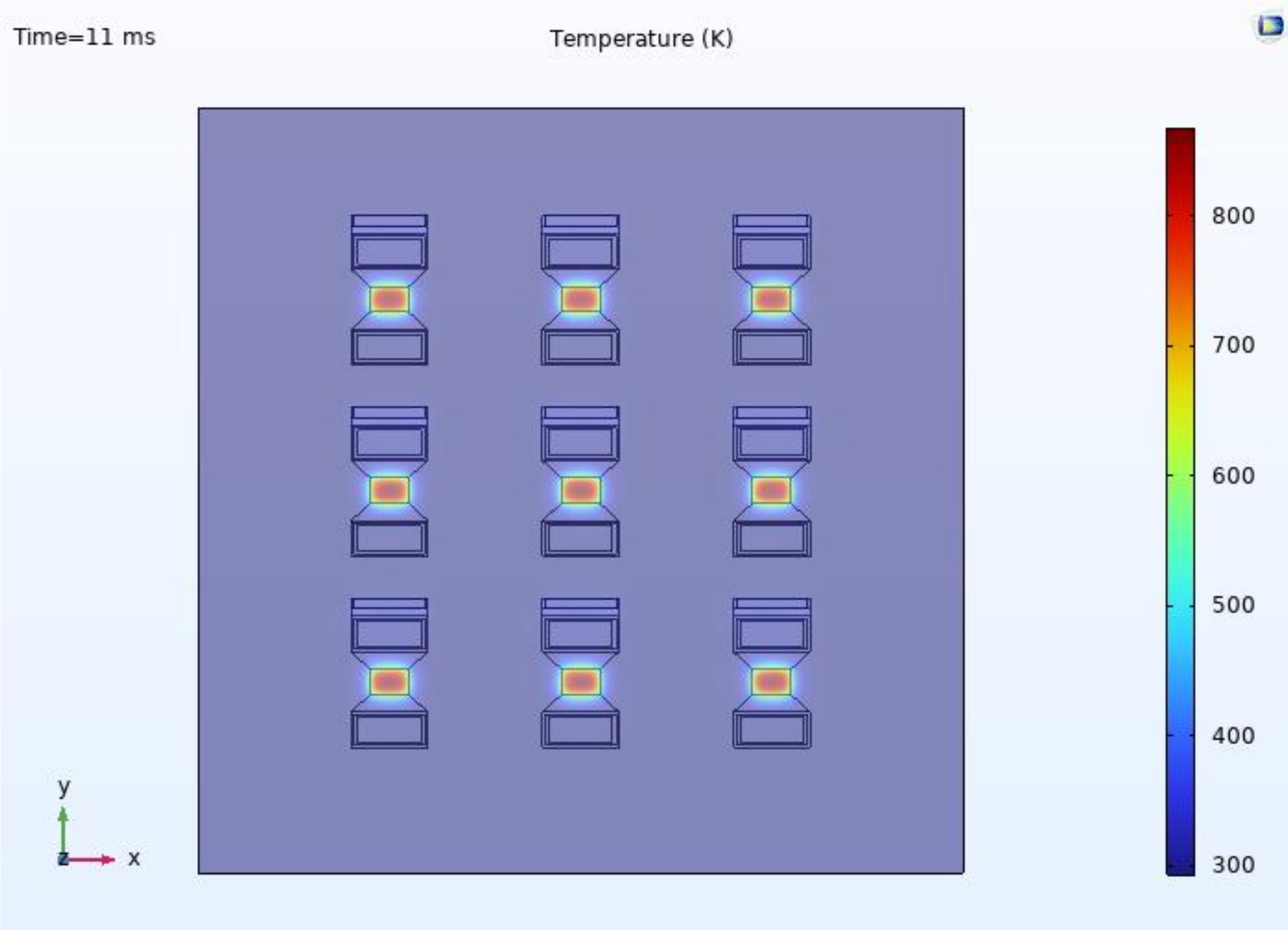

Below, an example image of one of the microheaters during processing is shown. Specifically, this image was collected right after the silicon nitride deposition and before etch back or aperture deposition. The COMSOL simulations show that the 500 K region barely extends outside the 40 x 60 um rectangle of medium doped silicon, even after 11 ms of heating for crystallization. As such, for the current design, significant thermal cross-talk is not expected to exist to the level of preventing switching of the devices or have interference between devices. While future designs aiming to increase fill factor may reach the regime where thermal cross-talk is significant, assuming that the metal layers are able to carry the required current, a more likely bottleneck in the current design (which can be addressed with more metal layers), simultaneous switching of all microheaters is not expected to be restricted by the quenching rate of the heaters.

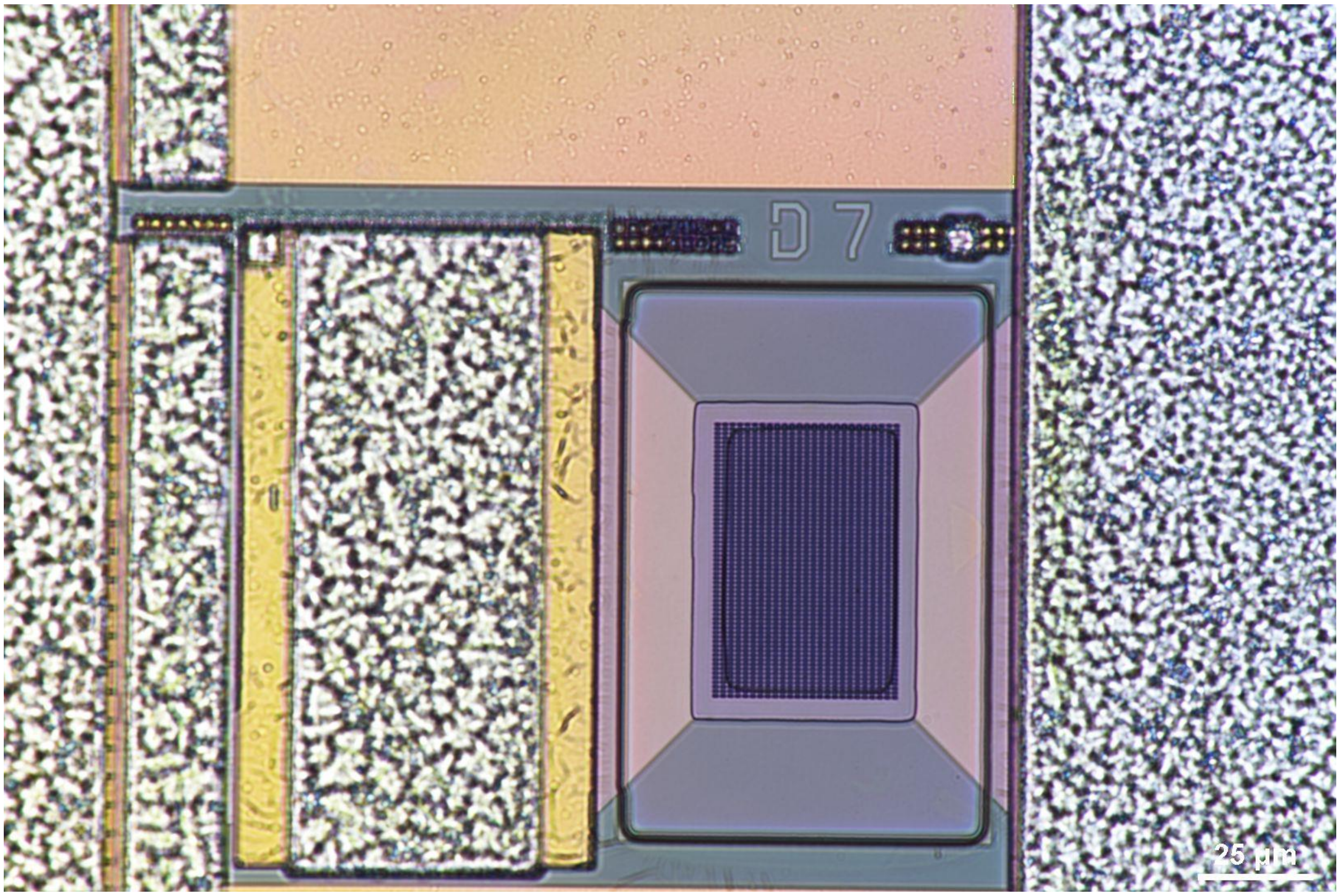

e. Since PCM switching is thermally driven, experimental characterization or simulation of thermal cross-talk between neighboring pixels would be important for evaluating large-array operation.

In the current design, the thermal cross-talk between devices is negligible.

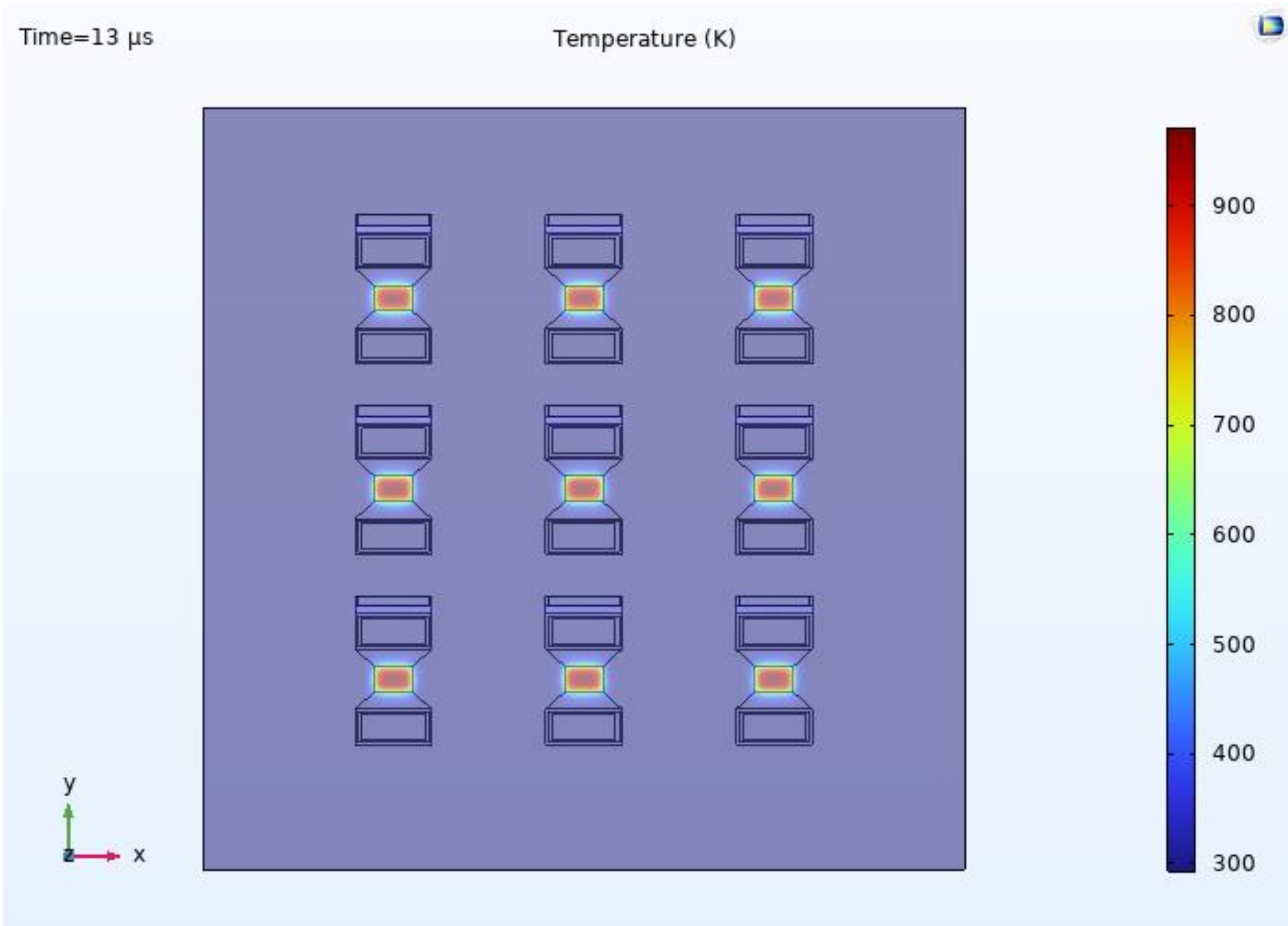
Time=13 µs
Temperature (K)
900
800
700
600
500
400
300
y
z
x

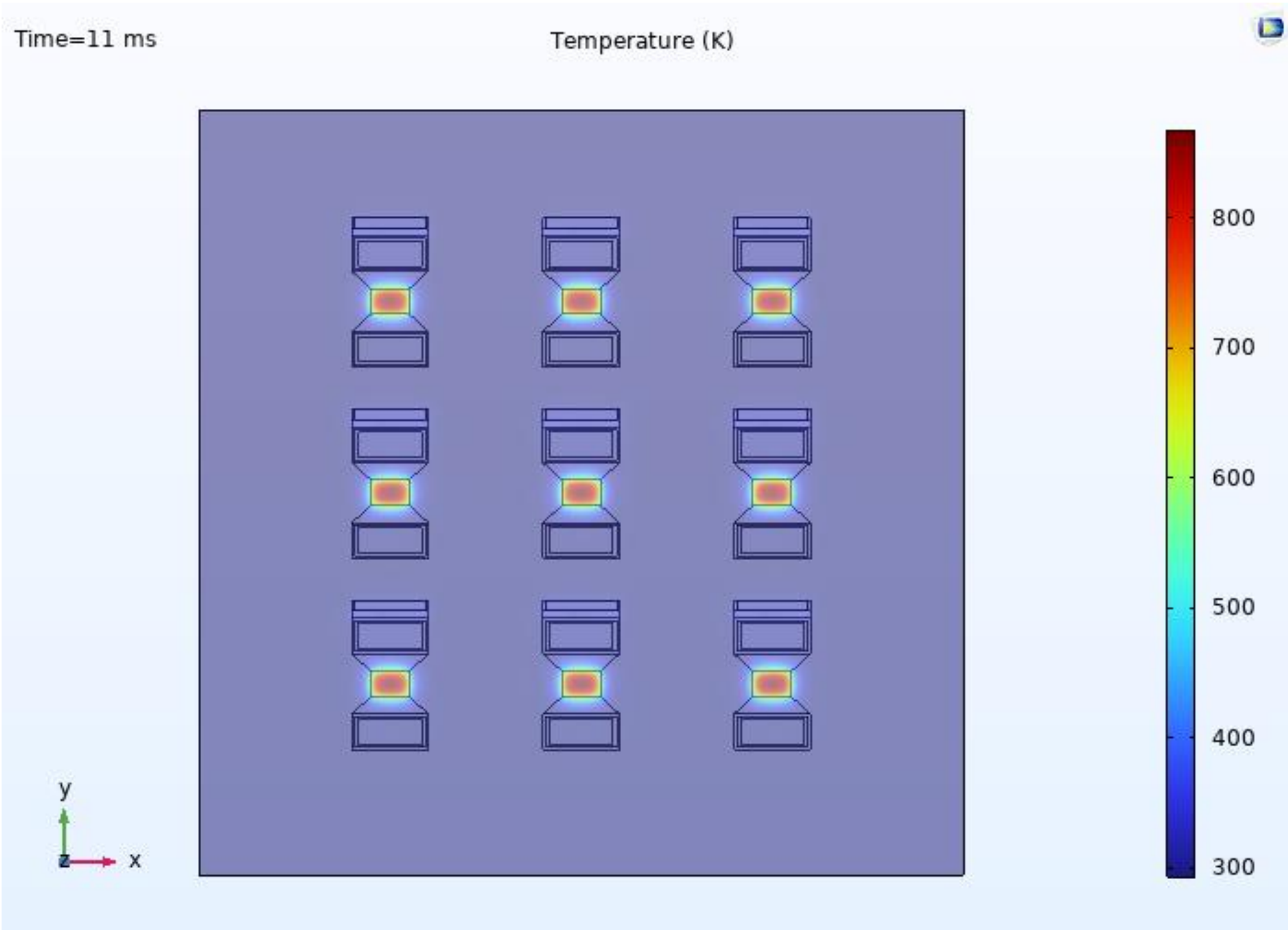

f. Although framed as an SLM, the demonstrated functionality is essentially pixelated amplitude modulation, without demonstration of phase control or wavefront-shaping capabilities. Although this critique may appear purely semantic, clarifying this distinction would improve the positioning of the work, as SLM is often used for amplitude, phase, and combined phase-amplitude modulation.

We thank the reviewer for this comment. Spatial light modulators are sometimes defied as “ a device that imposes variations in the amplitude, phase, or both, of a light beam.” (Restaino, S.R., 2015. Introduction to liquid crystals for optical design and engineering. Chapter 5, Society of Photo-Optical Instrumentation Engineers (SPIE). https://doi.org/10.1117/3.2197135.ch5). Digital micromirror devices are one form of spatial light modulators and their operation is fundamentally amplitude only operation. “Digital micro-mirror devices (DMDs) have recently emerged as practical spatial light modulators (SLMs) for applications in photonics, primarily due to their modulation rates,…” (Turtaev, S., Leite, I.T., Mitchell, K.J., Padgett, M.J., Phillips, D.B. and Čižmár, T., 2017. Comparison of nematic liquid-crystal and DMD based spatial light modulation in complex photonics. *Optics express*, *25*(24), pp.29874-29884.).

In another work, there is a commercial example of DMD that is referred to as SLM "For this, binary Fourier holograms were synthesized and images were numerically and optically reconstructed using digital micromirror (DMD) spatial light modulator (SLM) Texas Instruments Discovery 4100." (Goncharov, D., Starikov, R., Ponomarev, N. and Molodtsov, D., 2020. Implementation of Fourier holograms using high-performance DMD SLM. *Procedia Computer Science*, *169*, pp.558-563.)

As an example in patents, the following statement can be found in patent US 7,224,335 B2 "This invention relates to image display systems that employ so-called reflective spatial light modulators (SLMs) Such as those in the form of digital micro-mirror devices (DMDs). " Gibbon, M.A., Read, S., Zhou, S.Z. and Adkins, S., Imax Corp, 2007. *DMD-based image display systems*. U.S. Patent 7,224,335., implying that DMDs are a form of SLM.

We have clarified in the manuscript that our devices are operating as amplitude-only modulation.

"Here we introduce a scalable 2D architecture based on phase-change materials (PCMs) integrated metasurfaces and apply it to realize the first transmissive mid-infrared (mid-IR) amplitude-only spatial light modulator (SLM)."

Answer to reviewer:

Reviewer #4 (Remarks to the Author):

The revised manuscript presents a demonstration of a pixel-level, electrically addressable, transmissive metasurface operating in the mid-infrared. The combination of PCM-based non-volatile switching, selector-enabled crossbar addressing, and foundry-compatible BEOL integration is a notable contribution toward scalable programmable metasurfaces.

In my perspective, the authors have addressed all the concerns raised in the previous review round, and the manuscript has improved in clarity and completeness. The authors' commentary on the measurement limitation, parasitic effects, scalability, plausible device operation, and work positioning, answers to all my prior queries.

I would, however, encourage one more the authors to slightly refine the wording of the novelty claims in the initial paragraph of the Discussion/Conclusion, where multiple "first-of-their-kind" statements are presented. The current phrasing give the impression of orthogonal advances (lines 199-203), whereas the novelty lies in the combination of mid-IR transmissive operation, selector-enabled crossbar scalability, and foundry-compatible BEOL integration (as the authors remark elsewhere). A minor adjustment to reflect this integrated contribution would improve precision and avoid ambiguity. This is however a matter of clarity, rather than substance; it does not affect the significance of the results.

Overall, the manuscript is suitable for publication.

We thank the reviewer for the comments and support for publication. We thank the reviewer also for the prior comments that helped improve clarity and completeness of the manuscript.

We altered the text as "This work establishes and integrates multiple first-of-their-kind advances in active metasurface technology."

The statements in the text are intersections of criteria, in which we tried to restrict the scope of each phrase for precision, noting these firsts, to the extent of our knowledge.

Specifically, 1 - 2D 2- electrically addressable 3 - active 4 - metasurface with 5 - a scalable architecture, or 1 - transmissive 2 -mid-IR 3 -SLM, or 1 - PCM-based 2 - metasurface fabricated using 3 - a standard silicon foundry process with 4 - full BEOL integration.
